# A Zika virus protein expression screen in *Drosophila* to investigate targeted host pathways during development

**DOI:** 10.1242/dmm.050297

**Published:** 2024-02-28

**Authors:** Nichole Link, J. Michael Harnish, Brooke Hull, Shelley Gibson, Miranda Dietze, Uchechukwu E. Mgbike, Silvia Medina-Balcazar, Priya S. Shah, Shinya Yamamoto

**Affiliations:** ^1^ Department of Neurobiology, University of Utah, Salt Lake City, UT, 84112, USA; ^2^ Howard Hughes Medical Institute, Baylor College of Medicine (BCM), Houston, TX, 77030, USA; ^3^ Department of Molecular and Human Genetics, BCM, Houston, TX, 77030, USA; ^4^ Jan and Dan Duncan Neurological Research Institute, Texas Children's Hospital, Houston, TX, 77030, USA; ^5^ Postbaccalaureate Research Education Program (PREP), Houston, TX, 77030, USA; ^6^ Department of Chemical Engineering, Department of Microbiology and Molecular Genetics, University of California, Davis, CA, 95616, USA; ^7^ Department of Neuroscience, BCM, Houston, TX, 77030, USA; ^8^ Development, Disease Models & Therapeutics Graduate Program, BCM, Houston, TX, 77030, USA

**Keywords:** *Drosophila*, Zika virus, Microcephaly, Virus-host targets, Degeneration

## Abstract

In the past decade, Zika virus (ZIKV) emerged as a global public health concern. Although adult infections are typically mild, maternal infection can lead to adverse fetal outcomes. Understanding how ZIKV proteins disrupt development can provide insights into the molecular mechanisms of disease caused by this virus, which includes microcephaly. In this study, we generated a toolkit to ectopically express ZIKV proteins *in vivo* in *Drosophila melanogaster* in a tissue-specific manner using the GAL4/UAS system. We used this toolkit to identify phenotypes and potential host pathways targeted by the virus. Our work identified that expression of most ZIKV proteins caused scorable phenotypes, such as overall lethality, gross morphological defects, reduced brain size and neuronal function defects. We further used this system to identify strain-dependent phenotypes that may have contributed to the increased pathogenesis associated with the outbreak of ZIKV in the Americas in 2015. Our work demonstrates the use of *Drosophila* as an efficient *in vivo* model to rapidly decipher how pathogens cause disease and lays the groundwork for further molecular study of ZIKV pathogenesis in flies.

## Teaser

**Editor's choice:** Expression of Zika virus proteins in *Drosophila* causes tissue-specific phenotypes, suggesting that they interact or inhibit specific host pathways during development to cause disease.

## INTRODUCTION

Zika virus (ZIKV) is a small RNA virus that gained notoriety for its ability to cause a spectrum of congenital abnormalities dubbed congenital Zika syndrome (CZS) (da Silva Pone et al., 2018). This disorder occurs in infants after infection *in utero* and can include microcephaly, ocular abnormalities, congenital contractures and hypertonia that restricts body movements, with the possibility of additional defects arising later in development (Medina and Medina-Montoya, 2017). Some children without physical findings at birth also develop adverse neurological defects, developmental delays and microcephaly at a later point (Aragao et al., 2017; Bertolli et al., 2020; Carvalho et al., 2019; Rice et al., 2018; van der Linden et al., 2016), indicating that ZIKV has much broader effect on neurodevelopment and function than initially appreciated. Microcephaly, or reduced head size typically associated with smaller brain size, is one of the most severe outcomes in CZS and is associated with cognitive and neurological disorders. Given the severe outcomes associated with ZIKV infection *in utero*, it is critical to understand how ZIKV disrupts brain development.

In adults, ZIKV infection can be mild with rash, fever or muscle pain. However, some patients develop Guillain–Barré syndrome, an autoimmune disorder in which the body attacks its own peripheral nervous system. Patients affected by Guillain-Barré syndrome often experience muscle weakness or severe paralysis, but most people recover from this condition (Cao-Lormeau et al., 2016). In addition, ZIKV has also been linked to severe neurological disease in adults (Mehta et al., 2018). This includes meningoencephalitis (Carteaux et al., 2016), sensory neuropathy (Medina et al., 2016) and seizures (Asadi-Pooya, 2016). As the spectrum of disease is wide, a greater understanding of how ZIKV affects mature tissues in addition to developing tissues would be beneficial for patient treatment.

In addition to clinical evidence, animal models have also shown that ZIKV can have long term impact on nervous system function. For example, ZIKV infections in mice led to long term neuropathological effects that persisted into adulthood (Nem de Oliveira Souza et al., 2018), and postnatal infections in rhesus macaques resulted in behavioral, motor and cognitive deficits associated with abnormalities in brain structure (Raper et al., 2020). Thus, it is likely that this pathogen affects multiple molecular pathways that are associated with development and neural function, resulting in the wide spectrum of phenotypes associated with ZIKV infection during development or in adulthood.

**Fig. 1.**
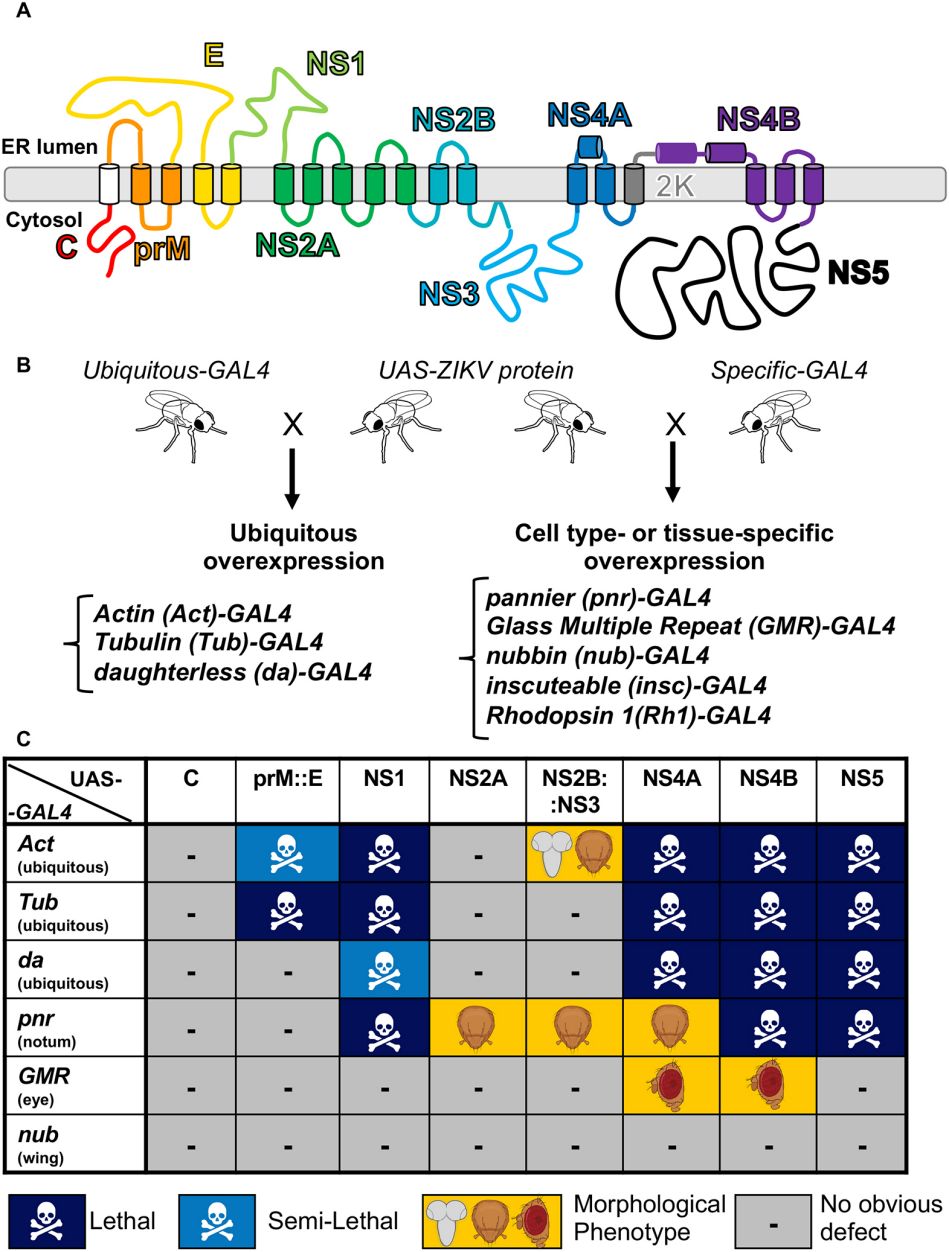
**ZIKV proteins cause scorable phenotypes upon overexpression in *Drosophila.*** (A) Graphical diagram of the Zika virus (ZIKV) polyprotein, which contains three structural and seven non-structural proteins at the endoplasmic reticulum (ER) membrane. (B) Diagram of the crossing scheme using which we crossed a fly containing a ZIKV protein under the control of a UAS element to another fly containing a GAL4 driver. The resulting fly expressed the ZIKV protein and was scored for phenotypes. (C) Table showing the phenotypes of the F1 generation resulting from a cross reared at 25°C. Dark blue represents lethality. Light blue represents semi-lethality, where less than 75% of expected Mendelian ratios are observed. Yellow indicates a morphological defect; the specific tissues affected are noted by the illustration (brain, thorax bristle and eye). The lethal stage for some crosses is indicated in Table S2.

The mechanism by which ZIKV causes disease, especially microcephaly, has become a major research topic since the declaration of the ZIKV epidemic in 2016 (https://www.who.int/groups/zika-virus-ihr-emergency-committee). Current data suggest that molecular mechanisms of ZIKV-induced microcephaly is mediated by inhibition of multiple host pathways by multiple ZIKV proteins. The genome of ZIKV is ∼10 kb and encodes three structural proteins [the capsid protein (hereafter Capsid or C), precursor membrane protein (prM) and envelope protein (E)] and seven nonstructural proteins (NS1, NS2A, NS2B, NS3, NS4A, NS4B and NS5) (Fig. 1A). In one study, Liang et al. (2016) found that ZIKV NS4A and NS4B suppress Akt-mTOR signaling in human fetal neural stem cells, inhibiting neurogenesis and upregulating autophagy. In another study, by individually expressing each ZIKV protein in the mouse cortex *in vivo*, Yoon et al. (2017) discovered that NS2A inhibits neurogenesis and proliferation of neural stem cells by disrupting adherens junctions. Protein interaction studies in human induced pluripotent stem cells (iPSCs) found that Capsid interacts with Dicer, and further functional studies demonstrated that ZIKV inhibits Dicer and host microRNA biogenesis, which, in turn, disrupts neurogenesis (Zeng et al., 2020). By taking a systems-level approach in humans to identify potential targets of ZIKV, we previously found that the ZIKV protein NS4A physically interacts with ANKLE2, a protein linked to a rare Mendelian form of congenital microcephaly (Khan et al., 2017; Yamamoto et al., 2014), in human cells (Shah et al., 2018). Using *Drosophila melanogaster*, we further showed that the expression of NS4A caused reduced brain volume *in vivo*, which could be rescued by co-expression of wild-type human ANKLE2 (Link et al., 2019; Shah et al., 2018). These results show that NS4A binds to and inhibits ANKLE2, providing a compelling mechanism as to how ZIKV infection causes microcephaly. Particularly, this last study provides an example of how *Drosophila* can be utilized as a model system to elucidate mechanisms of infectious diseases (Harnish et al., 2021).

In this study, to better understand the mechanisms of ZIKV pathogenesis, we generated a comprehensive toolkit of transgenic flies that allow expression of ZIKV proteins using the GAL4/UAS binary expression system (Brand and Perrimon, 1993). In addition to generating transgenic lines that allow the expression of ZIKV proteins from the Puerto Rican strain associated with the recent epidemic of CZS (Lanciotti et al., 2016), we also generated several transgenic lines that allow expression of proteins from a less pathogenic viral strain isolated in Cambodia prior to the ZIKV epidemic (Liu et al., 2017; Xia et al., 2018; Yuan et al., 2017) to assess whether the *Drosophila* resource can be used to study how virus evolution impacts pathogenesis. Using these tools and performing a series of phenotypic assays related to the nervous system, we show that *Drosophila* can deepen our understanding of ZIKV-induced developmental and post-developmental neuronal symptoms in human.

## RESULTS

### Expression of most ZIKV proteins or protein complexes causes lethality or morphological phenotypes when ectopically expressed in *Drosophila*

To determine how ZIKV proteins might hijack host pathways to cause disease, we sought to express each protein in a wide variety of tissues throughout development and in the adult fly using the GAL4/UAS system (Fig. 1B). To generate transgenic flies, we used sequence information from the PRVABC-59 strain isolated in Puerto Rico (Lanciotti et al., 2016) and synthesized and cloned individual protein-coding sequences into a UAS transgenic vector (pGW-HA.attB) (Bischof et al., 2013) using Gibson assembly (Gibson et al., 2009). The ZIKV genome is translated as one polyprotein and subsequently cleaved at established viral and host protease cleavage sites (Fishburn et al., 2022). We therefore designed expression constructs based on the major individual proteins formed following polyprotein processing. Each protein of interest was tagged with a C-terminal 3×HA tag for visualization and expression analysis, and careful consideration was taken to include proper signal sequences for transmembrane proteins (Shah et al., 2018). Transgenic strains were generated using the ɸC31 transgenesis technique, allowing integration of all constructs into an identical genomic locus of the fly at the established *attP*-docking site (VK37) at 22A3 on the second chromosome (Venken et al., 2006). We generated transgenic lines that allow expression of six proteins encoded in the ZIKV genome as single proteins: Capsid (C), NS1, NS2A, NS4A, NS4B and NS5. We also generated a construct that combines prM with E (prM::E) and NS2B with NS3 (NS2B::NS3). The former transgene was generated because prM is known to act as a co-chaperone for proper folding of E, and the two proteins form heterodimers that are critical for flaviviruses to form infectious particles (Yu et al., 2008). The latter transgene was generated because NS2B and NS3 form a heterodimer, which functions as a key viral protease that processes the viral precursor polyprotein (Falgout et al., 1991). In addition, as there are proteolytic cleavage sites on both sides of the short peptide 2K located between NS4A and NS4B (Sun et al., 2017), we generated constructs for NS4A and NS4B with and without 2K (NS4A, NS4A::2K, 2K::NS4B and NS4B). We also generated constructs for NS1 and prM::E with altered amino acids that have been implicated in infectability or pathogenicity. These constructs express NS1^cam^ and prM::E^cam^, which carry single amino acid changes that are found in a less pathogenic strain of ZIKV, FSS13025, isolated in Cambodia (Liu et al., 2017).

NS1^cam^ carries an alanine (A) in position 188 instead of the valine (V) in the NS1 in the Puerto Rican strain (NS1^pr^). We generated this transgene because the p.A188V mutation in NS1 increases the infectivity of ZIKV in mosquitos (Liu et al., 2017) and inhibits interferon β production in human cells (Xia et al., 2018). prM::E^cam^ carries a serine (S) in position 139 of the E protein instead of the asparagine (N) in E protein in the the Puerto Rican strain (E^pr^). This construct was generated because a p.S139N mutation increased infectability of the virus in human and mouse neural progenitor cells and caused a more severe microcephaly phenotype in mouse models (Yuan et al., 2017). In total, we generated 12 transgenic lines (Table S1).

**Fig. 2.**
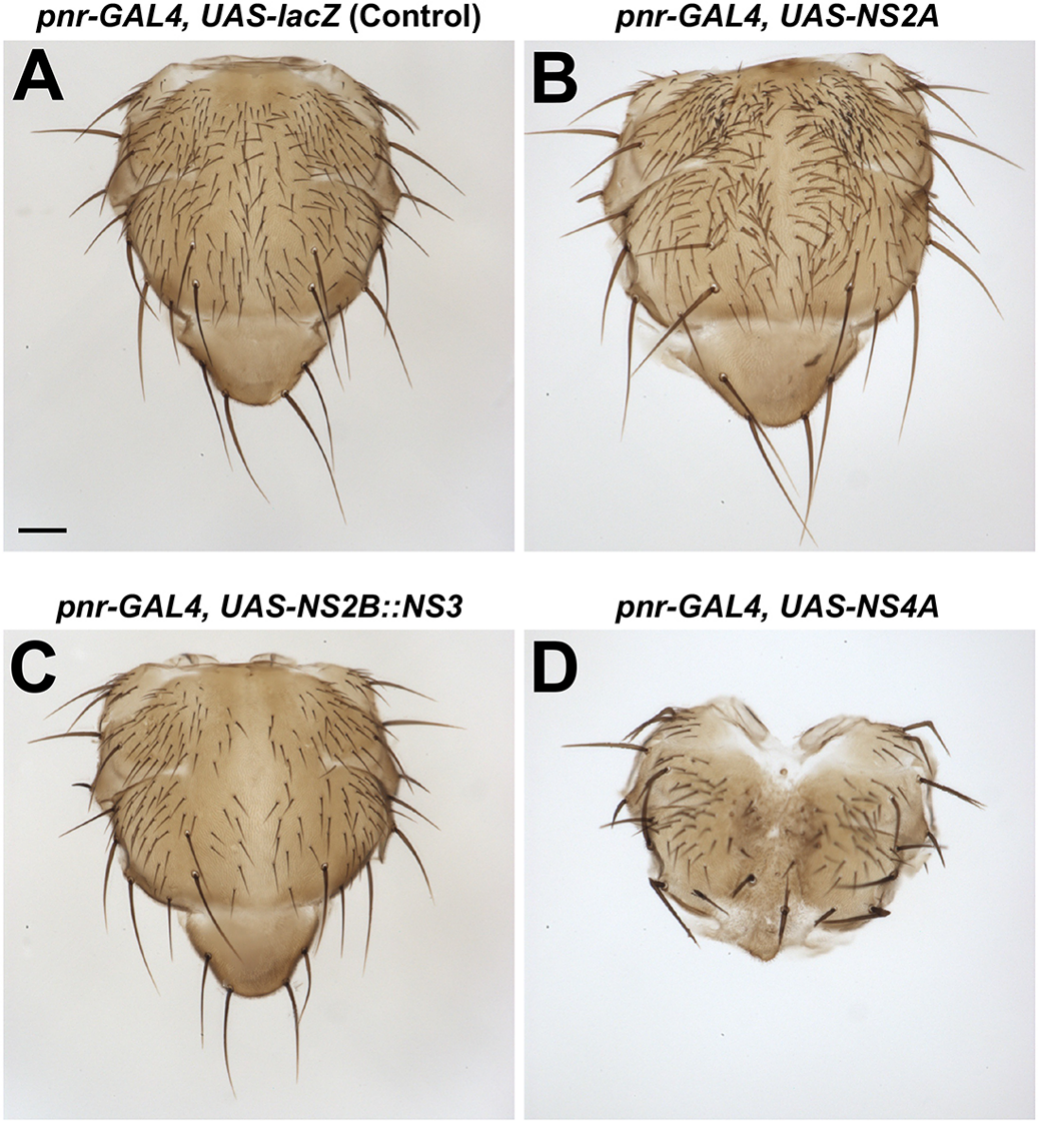
**Expression of ZIKV proteins in the notum causes bristle and split thorax phenotypes.** (A) Control notum showing no morphological defects. (B) Expression of NS2A in the dorsocentral notum caused a supernumerary bristle phenotype. (C) Notum from animals with NS2B::NS3 expression (*pnr-GAL4*, *UAS-NS2B::NS3*) showed bristle loss. (D) A rare escaper notum from animals with NS4A expression demonstrates a split thorax phenotype and bristle defects. For each case, the penetrance of phenotype was 100%. All crosses were carried out at 29°C. Images are representative of at least 100 animals per genotype. Scale bar: 0.1 mm.

**Fig. 3.**
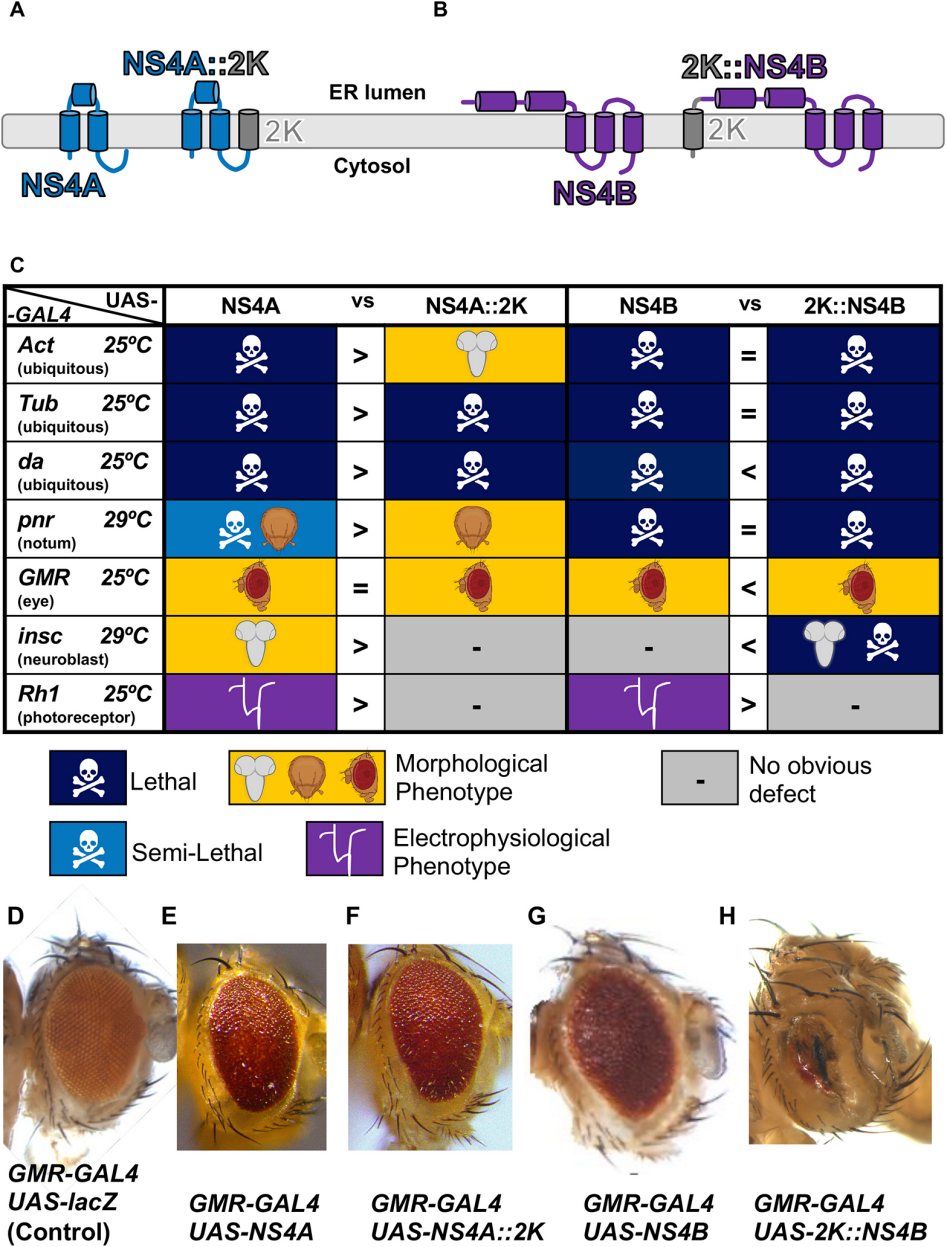
**2K peptide alters NS4A and NS4B phenotypes.** (A,B) Graphical diagram of the ZIKV NS4A and NS4A::2K peptides (A) and the 2K::NS4B and NS4B peptides (B) at the ER membrane. Both NS4A and NS4B can be found with the 2K peptide linker region. (C) Table showing the phenotypes of the F1 generation resulting from a cross reared with indicated drivers at 25°C or 29°C. Dark blue represents lethality. Light blue represents semi-lethality, where less than 75% of expected Mendelian ratios are observed. Yellow indicates a morphological defect; specific tissues affected are noted by the illustration (brain, thorax bristle and eye). Purple indicates an electrophysiological phenotype. The ‘vs’ column denotes whether one variant is more severe (> or <) or both have equal severity (=). The lethal stage for some crosses is indicated in Table S2. (D-H) Eye phenotypes as a result of *GMR-GAL4* expression of control lacZ (D), NS4A (E), NS4A::2K (F), NS4B (G) and 2K::NS4B (H). Note that in general, the 2K peptide decreased the effect of NS4A but enhanced the phenotypes caused by NS4B. In each case, the penetrance of phenotype was 100%. Images are representative of at least 100 animals per genotype.

First, we crossed eight transgenic lines (*UAS-C*, *UAS-prM::E*, *UAS-NS1*, *UAS-NS2A*, *UAS-NS2B::NS3*, *UAS-NS4A*, *UAS-NS4B* and *UAS-NS5*) from the Puerto Rican strain to a variety of GAL4 lines to express each viral or fusion protein in a variety of tissues at different developmental timepoints. This would allow us to cast a wide net to assess host pathways that ZIKV might hijack during infection and disease progression. We tested three ubiquitous drivers with variable strengths [*α-Tubulin at 84B* (*αTub84B* or *Tub*)*-GAL4*, *Actin* (*Act5C* or *Act*)*-GAL4* and *daughterless* (*da*)*-GAL4*] and three tissue-specific drivers [*nubbin* (*nub*)*-GAL4* for the wing, *pannier* (*pnr*)*-GAL4* for the dorsal thorax and glass multiple reporter (*GMR*)*-GAL4* for the eye] to assess the functional consequences of overexpressing the ZIKV proteins on viability and gross morphology. We found that seven out of eight lines (prM::E, NS1, NS2A, NS2B::NS3, NS4A, NS4B and NS5) caused scorable phenotypes with at least one of these drivers (Figs 1C and 2, Table S1). Ubiquitous expression revealed that some viral proteins (prM::E, NS1, NS4A, NS4B and NS5) caused lethality, suggesting that these proteins likely impact essential cellular pathways. The lethal stage induced by some ZIKV proteins is presented in Table S2. Overexpression of N2A caused increase in mechanosensory bristles, whereas NS2B::NS3 overexpression caused reduction of mechanosensory bristles on the dorsal thorax of the fly, indicating that these proteins affect pathways that are required for peripheral nervous system development (Figs 1C and 2B-C) (Schweisguth, 2015; Schweisguth et al., 1996). Overexpression of NS4A using *pnr-GAL4* caused a dorsal thorax closure defect (Figs 1C and 2D), which is a phenotype often seen when cell migration or cell communication through JNK and TGF-β/BMP signaling is defective (Agnès et al., 1999; Martin-Blanco et al., 2000). Overexpression of NS4A or NS4B using *GMR-GAL4* caused a rough eye morphology phenotype (Figs 1C and 3D-H), suggesting a developmental defect of the compound eye, which can be caused by disruption of many pathways and biological processes (Kumar, 2001; Thomas and Wassarman, 1999). In contrast, although the development of the wing depends on many signaling pathways and cellular processes (Bier, 2005), none of the lines examined, including those expressing the ZIKV proteins that cause lethality when overexpressed ubiquitously, caused an obvious wing morphological defect when the ZIKV proteins were expressed using *nub-GAL4*. Therefore, ZIKV proteins appear to affect specific proteins, pathways or cellular processes to induce a phenotype upon overexpression in flies in a context-specific manner, rather than through inducing a general cellular toxicity.

### The site of 2K peptide cleavage can affect the function of NS4A and NS4B

Between the NS4A and NS4B proteins, there is a linker sequence that is referred to as the 2K peptide (Fig. 1A) (Lin et al., 1993). The site between NS4A and 2K is cleaved by the viral NS2B::NS3 protease and the site between 2K and NS4B is cleaved by one or more host proteases, respectively (Sun et al., 2017). Because the two cleavage events occur sequentially (Lin et al., 1993) and 2K has been shown to alter NS4A function (Miller et al., 2007; Roosendaal et al., 2006), we tested whether expressing a fusion protein of NS4A and 2K (NS4A::2K) or 2K and NS4B (2K::NS4B) has a different effect than expressing these two proteins without this peptide (Fig. 3A,B).

Ectopic expression of NS4A without the 2K peptide caused multiple scorable phenotypes with diverse GAL4 lines, including lethality with *Act-GAL4*, *Tub-GAL4* and *da-GAL4* (Figs 1C and 3C). Expression with *GMR-GAL4* produced animals with rough eye phenotypes (Figs 1C and 3C,E) and *pnr-GAL4* expression led to dorsal thorax closure (Figs 1C, 2D and 3C). When we expressed NS4A::2K with the same GAL4 drivers, we observed mostly weaker phenotypes compared to those seen upon expression of NS4A alone (Fig. 3C,F). Expression of NS4A caused lethality in most animals with few escapers using *pnr-GAL4* at 29°C, but lethality was not observed when NS4A::2K was expressed using this driver and temperature. However, ubiquitous expression of NS4A::2K with *da-GAL4* caused lethality during pupal stages, which is in contrast to NS4A causing early larval lethality using the same driver. Similarly, viable flies that expressed NS4A::2K using *Act-GAL4* were observed, which were not seen when NS4A was expressed using the same driver. In summary, the 2K peptide can sometimes suppress the function of NS4A depending on the temperature, context and GAL4 drivers being used.

Similar to NS4A, ectopic expression of NS4B without the 2K peptide using multiple drivers affected viability and development. Expression of NS4B with all three ubiquitous drivers (*Act-GAL4*, *Tub-GAL4* and *da-GAL4*) caused lethality, whereas expression in the developing eye using *GMR-GAL4* induced rough eyes (Figs 1C and 3C,G).

To explore why *GMR-GAL4* expression of ZIKV NS4A and NS4B proteins caused rough eye phenotypes, we assessed cell division and cell death in developing eye discs. We counted the number of phospho-histone H3-positive cells, indicating a dividing cell, in the eye disc and found no significant changes (Fig. S1A-E). To mark cells undergoing apoptosis, we used activated Death Caspase 1 (Dcp1) and again found no significant changes at this stage (Fig. S1F-J). Therefore, the rough eye phenotype is likely be caused by other factors during morphogenesis or cell death occurring at later stages of development.

We also observed that addition of 2K affected the severity of phenotypes with some drivers (Fig. 3C,H). In three cases, the addition of 2K made the phenotype more severe. For example, using *da-GAL4*, expression of 2K::NS4B caused embryonic lethality, whereas expression of NS4B caused late larval lethality. Similarly, expression of 2K::NS4B caused a much more severe eye developmental defect compared to that seen upon expression of NS4B using *GMR-GAL4* (Fig. 3C,G,H). However, overexpression of 2K::NS4B did not change the lethality observed when NS4B was expressed using *Act-GAL4* or *Tub-GAL4* and made the phenotype caused by another driver weaker (*Rh1-GAL4*, see below). Therefore, although addition of the 2K peptide to NS4B enhances its function in some contexts, it can also be irrelevant or may decrease its function in other circumstances.

### Multiple ZIKV proteins reduce brain volume when expressed in *Drosophila*

**Fig. 4.**
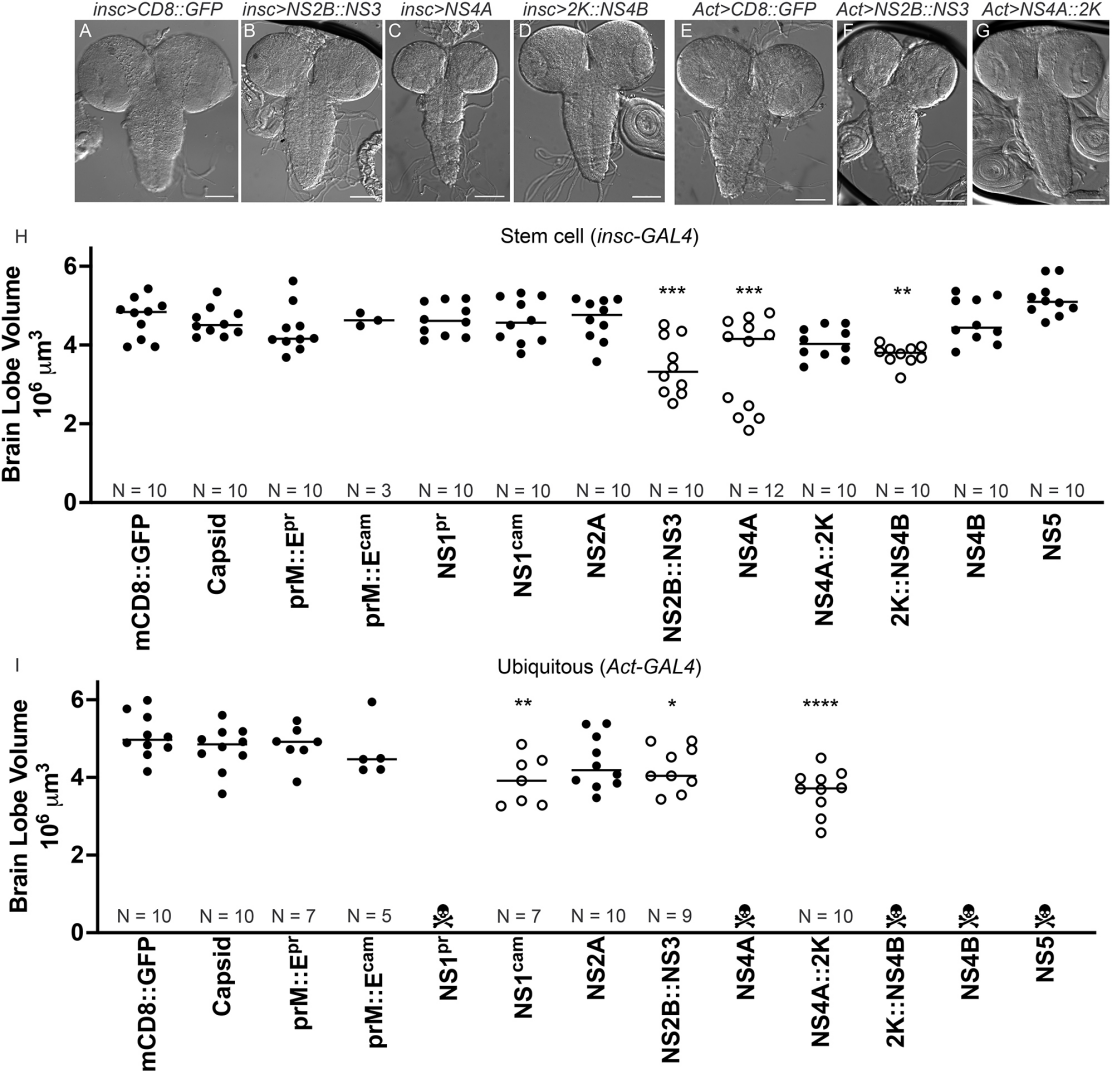
**Multiple ZIKV proteins cause microcephaly upon overexpression in *Drosophila.*** Expression of ZIKV proteins using either *insc*-*GAL4* at 29°C (A-D,H) or *Act*-*GAL4* at 25°C (E-G,I) caused microcephaly phenotypes. (A-G) Bright field images of brains from the indicated lines are shown. Scale bars: 100 µm. (H,I) Quantification of brain volume. Individual brain lobe volume measurements are plotted and the mean is represented by the line. Populations with smaller brain volumes and *P*<0.05 are open circles, whereas populations with *P*>0.05 are in closed circles. One-way ANOVA with multiple comparisons posttest compared to control (mCD8::GFP) was used to assess significance. Lethal crosses are indicated with a skull symbol and the number of animals for each condition are shown as N. *P<0.05; **P<0.01; ***P<0.001; ****P<0.0001.

As ZIKV infection is associated with severe microcephaly, we set out to define the set of viral proteins that might target important processes during brain development. Previously, we showed that expression of ZIKV NS4A::2K using ubiquitous (*Act-GAL4*) drivers or drivers specific to neural stem cells and their progeny [*inscuteable* (*insc*)*-GAL4)*] reduces the volume of third instar larval brains (Link et al., 2019; Shah et al., 2018). To determine whether other transgenes also have similar biological functions, we expressed each protein or fusion protein with *Act-GAL4* or *insc-GAL4*. Upon ubiquitous expression with *Act-GAL4* at 25°C, NS1, NS4A, NS4B, 2K::NS4B and NS5 caused lethality prior to the late third instar larval stage, so brains could not be assessed (Fig. 4). Expression of NS4A::2K using *Act-GAL4* caused significant brain size reduction compared to that in controls overexpressing mCD8::GFP, a neutral transmembrane protein (Fig. 4G,I; one-way ANOVA and multiple comparisons posttest), whereas expression of NS2B::NS3 caused a milder but more significant brain volume defect (Fig. 4F,I). Using *insc-GAL4* at 29°C, we found that expression of NS2B::NS3, NS4A, NS4A::2K and 2K::NS4B caused significant brain volume defects (Fig. 4A-E,H).

To determine possible reasons why ZIKV proteins cause significant brain volume reduction, we assessed cell division and cell death in *insc*>NS2B::NS3-, *insc*>NS4A- and *insc*>2K::NS4B-expressing animals. We chose a single timepoint in late third instar development for these experiments and, as a result, they provide a snapshot in time in a cell type-specific population. We counted the number of phospho-histone H3-positive neuroblasts in the central brain and found a non-significant decrease with NS4A and a significant decrease with 2K::NS4B expression (Fig. S2A-D). To mark cells undergoing apoptosis, we use activated Dcp1 and found a non-significant increase in both NS2B::NS3 and NS4A (Fig. S2E-H). In summary, these data not only support previous observations that NS4A expression (with or without 2K) in *Drosophila* causes reduced brain volume by interacting with the ANKLE2 pathway (rescue experiment data shown in Fig. S3) (Link et al., 2019; Shah et al., 2018), but also identify NS2B::NS3 and 2K::NS4B as previously unreported negative regulators of brain size when expressed in *Drosophila*.

### Immunostaining of epitope tags attached to ZIKV proteins expressed in *Drosophila* neuroblasts reveal different patterns of subcellular localization

**Fig. 5.**
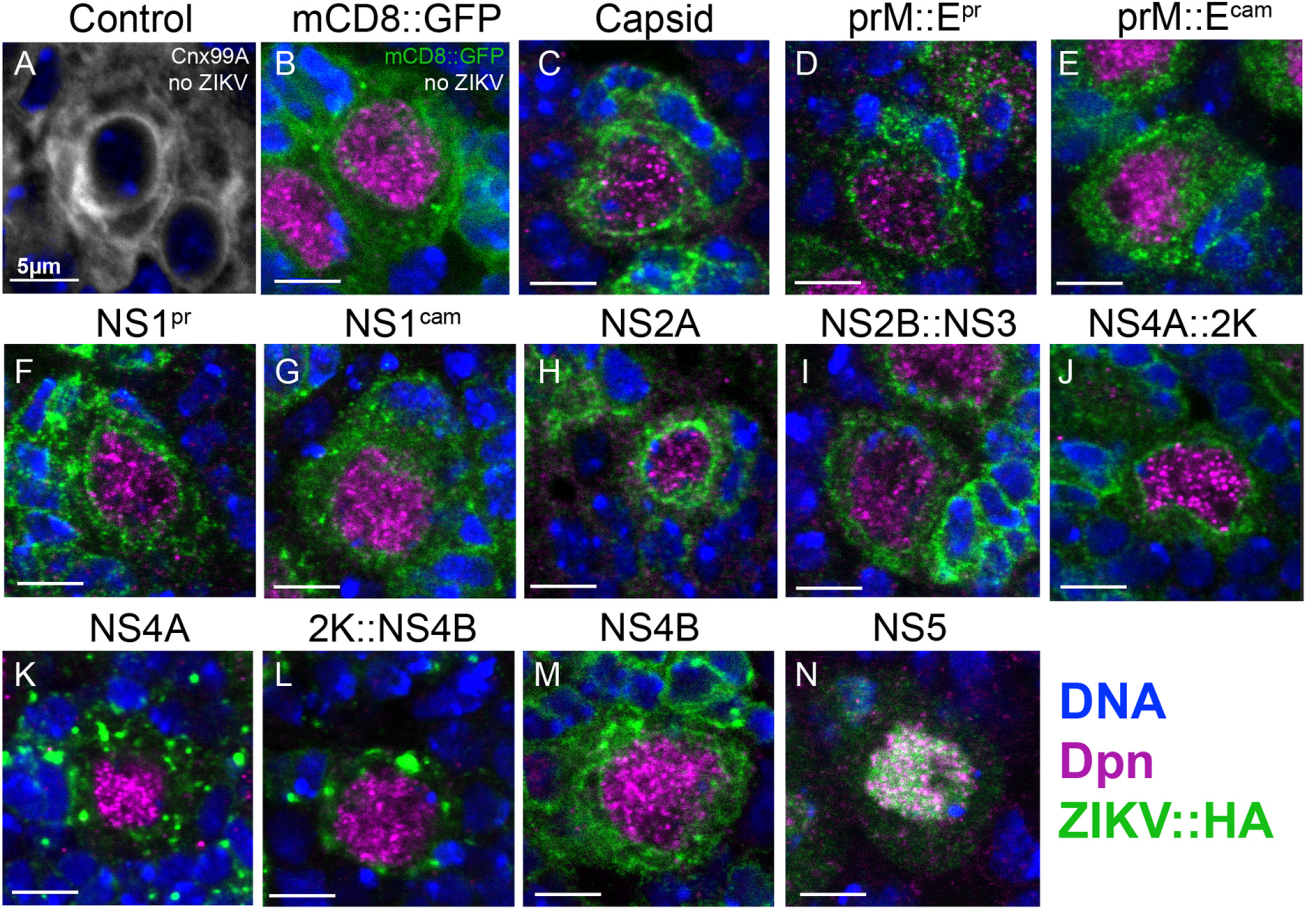
**Subcellular localization of ZIKV proteins differs when expressed in neuronal stem cells.** (A) Neural stem cells from wild-type animals stained with DAPI (blue) and Calnexin 99A (white) to highlight ER structure. (B) Animals expressing mCD8::GFP as a control with DAPI (blue), Dpn (magenta) and mCG8::GFP (green). (C-M) Neural stem cells from third instar larvae with *insc*-*GAL4* ZIKV protein expression stained for the C-terminal HA tag (green) to mark ZIKV proteins, Dpn (magenta) to indicate neuroblasts and DAPI to highlight DNA. Each panel represents a single stem cell in interphase. Scale bars: 5 µm. Note that the 2K peptide altered protein localization of NS4A and NS4B, whereas NS5 was localized in the nucleus. Images are representative of at least 20 animals per genotype.

ZIKV proteins are synthesized on the rough endoplasmic reticulum (ER), and replication and assembly of the virus takes place primarily in this organelle (Cortese et al., 2017). However, there are several studies that suggest that some viral proteins function in other subcellular organelles. For example, NS5 can function in the nucleus to modulate host cell division and gene expression (Conde et al., 2020; Kesari et al., 2020; Li et al., 2022; Petit et al., 2021; Zhao et al., 2021). We assessed the expression and subcellular localization pattern of these proteins using immunofluorescence staining of the C-terminal 3×HA tag and confocal microscopy. We dissected late third instar larval brains expressing a ZIKV transgene using *insc-GAL4* and immunostained them with antibodies against Deadpan (Dpn), a nuclear marker of neuroblasts (neural stem cells), and the HA tag, which labels each viral protein expressed (Fig. 5C-N). Many viral proteins [Capsid, prM::E^pr^, NS1^pr^, NS2A, NS4A::2K and NS4B] were localized in a pattern that was suggestive of ER localization (Fig. 5C,D,F,H,J,M), as indicated by the ER protein Calnexin 99A (Cnx99A) (Fig. 5A), consistent with previous observations in mammalian cells (Hou et al., 2017). Some proteins exhibited distinct patterns, including NS4A and 2K::NS4B that showed a punctate pattern (Fig. 5K,L), consistent with previous studies in mammalian cells (Miller et al., 2007; Roosendaal et al., 2006; Shah et al., 2018). NS5 showed nuclear localization (Fig. 5N), also consistent with previous studies in mammalian cells (Petit et al., 2021). It is interesting to note that the presence or absence of the 2K peptide on NS4A and NS4B, which can alter the phenotypic strength (Fig. 3), caused a change in the subcellular localization of these proteins (Fig. 5J-M).

### Expression of some ZIKV proteins in post-differentiated photoreceptor cells causes electrophysiological defects

**Fig. 6.**
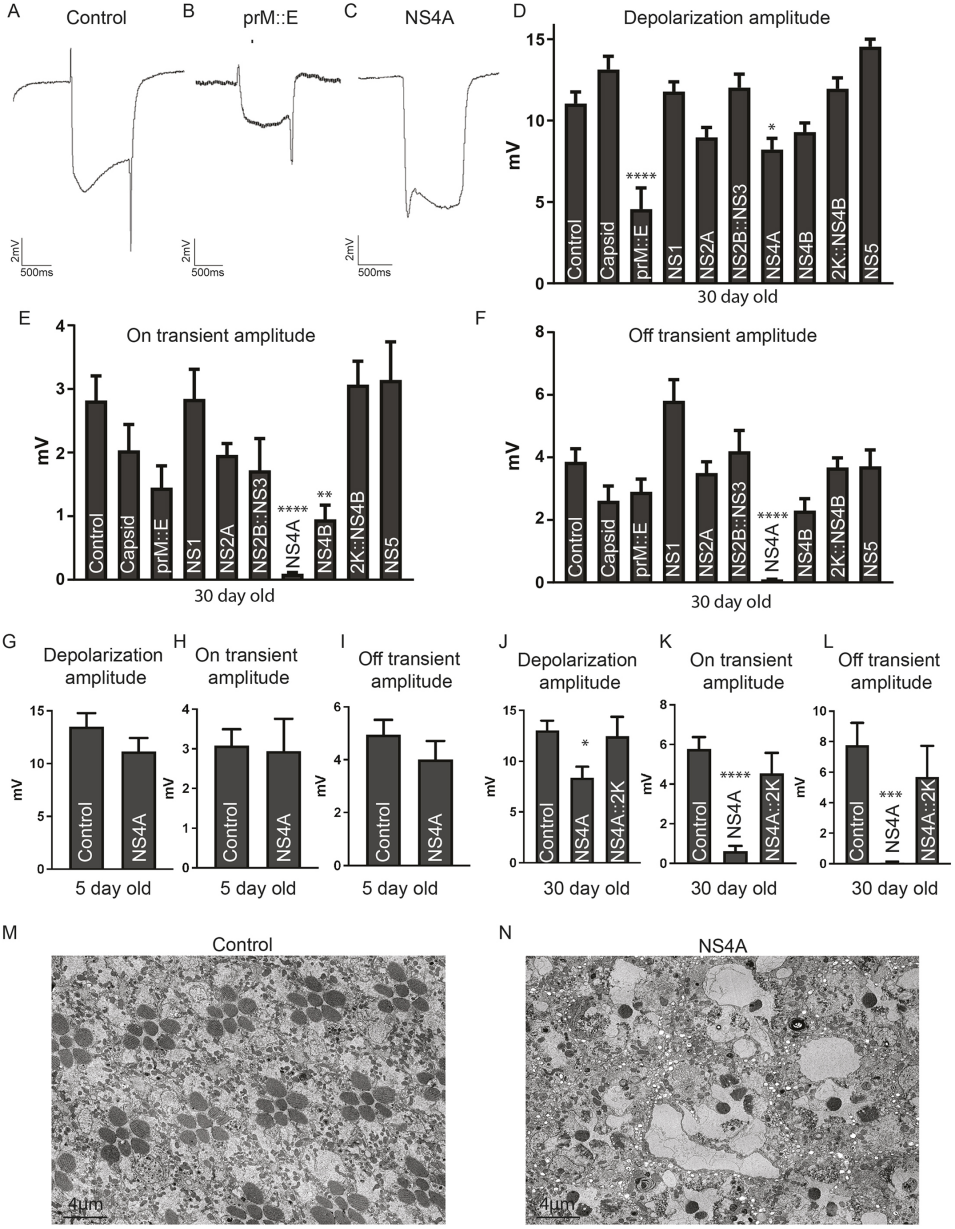
**Expression of some ZIKV proteins causes electrophysiological defects in the fly visual system.** (A-C) Representative electroretinograms (ERGs) from control (luciferase) (A) and prM::E (B) and NS4A (C) expressing animals 30 days after eclosion in a 12 h/12 h light/dark cycle. (D) Quantification of depolarization amplitude showed reduced depolarization amplitude in animals with neuronal expression of prM::E. (E,F) Quantification of on/off transient amplitudes showed loss of on- and off-transients with NS4A expression. (G-L) ERG depolarization amplitude (G,J) and on (H,K) and off (I,L) transient quantification of NS4A-expressing animals at 5 days after eclosion (G-I) and NS4A- and NS4A::2K-expressing animals at 30 days after eclosion (J-L). No defect was documented in 5-day-old animals (G-I), indicating that NS4A causes degenerative ERG defects over time. Comparisons between NS4A alone or with NS4A::2K (J-L) showed that only NS4A alone caused neuronal phenotypes at 30 days after eclosion. (M,N) Transmission electron microscopy of the retina of control animals expressing luciferase (M) or NS4A (N) with *Rh1-GAL4* in photoreceptors. Note that NS4A induced loss of photoreceptors, likely corresponding to the decrease in ERG amplitude over time. Images are representative of three animals per genotype. Scale bars: 4 µm. In D-L, data show the mean±s.e.m. One-way ANOVA with multiple comparisons posttest compared to control (luciferase) was used to assess significance. *P<0.05; **P<0.01; ***P<0.001; ****P<0.0001.

Adult humans infected with ZIKV can present with neurological symptoms (Mehta et al., 2018), whereas adult mice exposed to this virus can exhibit long-term neuropathological defects (Nem de Oliveira Souza et al., 2018). Thus, it is possible that ZIKV infects and impacts the function of the post-developmental nervous system. To test whether ZIKV proteins affect post-differentiated neurons, we expressed each protein in the fly photoreceptor cells using *Rhodopsin 1* (*Rh1*)-*GAL4* at 25°C. Photoreceptors in insects are light-sensory neurons that project axons into the lamina or medulla layers of the adult brain. *Rh1* [also known as *neither inactivation nor afterpotential E* (*ninaE*)] encodes a rhodopsin that is expressed in six out of eight photoreceptors (R1 to R6) that make up an ommatidia, the basic unit of a compound eye. The function of photoreceptor neurons can be assessed using electroretinogram (ERG) analyses, which are a cumulative measure of neuronal function in response to light from the *Drosophila* eye and brain (Ugur et al., 2016). Live flies were immobilized on glass slides with glue, a recording electrode was placed touching the eye, but not penetrating the tissue, and a reference electrode was placed behind the eye. The fly was acclimated to a dark room and a light was flashed on for a brief period, usually 1 s. When the light was flashed on, an ERG trace that consists of an on-transient spike followed by depolarization was recorded. When the light was turned off, an off-transient spike was observed, followed by repolarization of the potential to the baseline (Fig. 6). Depolarization/repolarization represents the phototransduction response of photoreceptors to light stimulus, whereas the on/off transient spikes correspond to signal transmission between pre- and post-synaptic neurons in the brain (Dolph et al., 2011). In mutants with degenerative phenotypes, the reduction in depolarization amplitude is often documented, which may be accompanied by reduction or loss of on/off transients (Deal and Yamamoto, 2018).

Adult animals expressing each viral protein were aged for 30 days post eclosion in a 12 h/12 h light/dark cycle at 25°C. For most ZIKV proteins, expression in photoreceptor cells did not affect ERG patterns. However, prM::E expression caused a strong decrease in ERG depolarization amplitude, indicating photoreceptor function defects (compare Fig. 6A to Fig. 6B,D).

In addition, expression of NS4A, but not NS4A::2K, caused loss of ERG on/off transient spikes in aged animals (Figs 3C and 6C,E,F,J-L). To determine whether this phenotype was age dependent, we tested the ERG on flies expressing NS4A using *Rh1-GAL4* that were only aged for 5 days post eclosion. Interestingly, we did not observe a difference in the on/off transient spikes or depolarization in these young animals (Fig. 6G-I). Therefore, the ERG defect caused by expression of NS4A is likely to be degenerative over time in adulthood rather than developmental or physiological in nature. Indeed, examination of the ultrastructure of the retina using transmission electron microscopy revealed that overexpression of NS4A causes significant reduction in the number of photoreceptors accompanied by many abnormal vacuoles (Fig. 6M,N). Finally, Expression of NS4B, but not 2K::NS4B, mildly decreased on transient amplitude (Figs 3C and 6D-F). Importantly, expression of other ZIKV proteins did not show these phenotypes (Fig. 6D-F), indicating that prM::E, NS4A and NS4B specifically inhibit the function or integrity of mature neurons.

### ZIKV proteins encoded by different strains show functional differences when tested in *Drosophila*

**Fig. 7.**
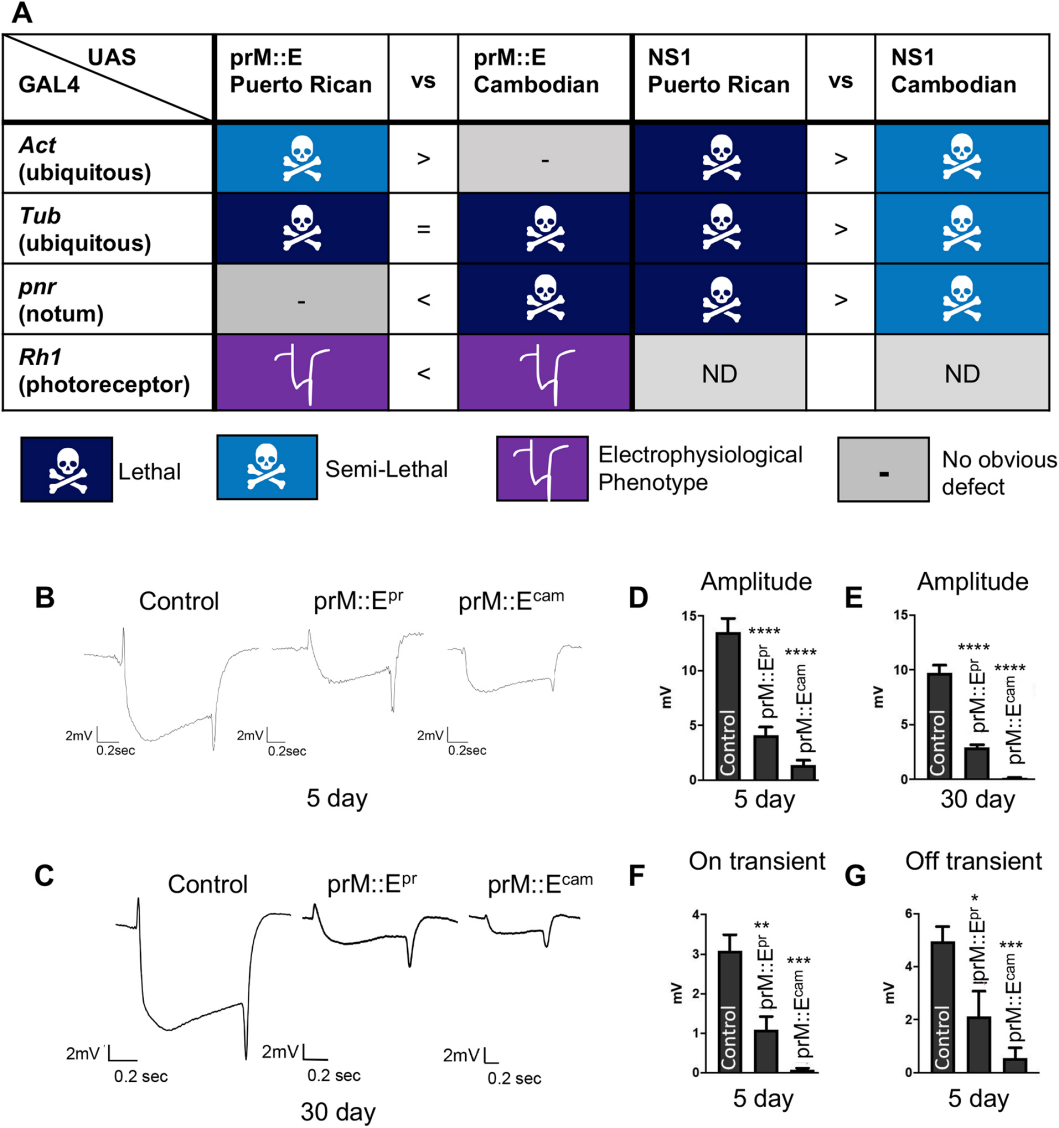
**ZIKV protein expression phenotypes for prM::E and NS1 are different based on the viral strain.** (A) Phenotypes when prM::E and NS1 from either the Puerto Rican or Cambodian strains of ZIKV were expressed by various GAL4 drivers. The ‘vs’ column denotes whether one variant is more severe (> or <) or both have equal severity (=). ND, not determined. (B,C) Representative ERG traces of control, prM::E^pr^- and prM::E^cam^-expressing animals at 5 days (B) or 30 days (C) after eclosion. (D,E) Depolarization amplitudes of 5-day-old (D) or 30-day-old (E) animals expressing luciferase (control), prM::E^pr^ or prM:E^cam^ show developmental ERG defects that are stable with age. The prM::E^cam^ phenotype is more severe than the prM::E^pr^ phenotype. (F,G) On transient (F) and off transient (G) amplitudes were also reduced with expression of prM::E^pr^ or prM:E^cam^ shown at 5 days after eclosion. Data show the mean±s.e.m. One-way ANOVA with multiple comparisons posttest compared to control (luciferase) was used to assess significance. *P<0.05; **P<0.01; ***P<0.001; ****P<0.0001.

Finally, we explored whether the *Drosophila* system could be used to test the functional consequences of mutations that were acquired in protein-coding genes during ZIKV evolution. The more recent Puerto Rican ZIKV strain, which is more closely related to strains responsible for recent outbreaks in the Americas, causes high rates of fetal microcephaly, similar to those seen in outbreaks in French Polynesia (Cauchemez et al., 2016). However, before these outbreaks, ZIKV infection was not associated with microcephaly. As the Cambodian strain was the last virus of the Asian lineage not associated with severe microcephaly (Duong et al., 2017), it is useful for comparative investigations. We focused on two mutations, one in NS1 and the other in E, as they had both been previously suggested to contribute to disease severity using other experimental systems (Wang et al., 2017; Xia et al., 2018; Yuan et al., 2017). The NS1 protein from the Puerto Rican strain (NS1^pr^) carries a p.A188V mutation compared to the NS1 protein from the more ancient Cambodian strain (NS1^cam^) (Delatorre et al., 2017). This mutation has been found to enhance the evasion of ZIKV from the host immune system based on studies performed in mice and human cells (Xia et al., 2018). When expressed using *Act-GAL4*, *Tub-GAL4* or *pnr-GAL4*, NS1^pr^ caused lethality (Figs 1C and 7A). However, expression of NS1^cam^ was only semi-lethal using the same drivers tested under the same conditions (Fig. 7A). This suggests that NS1^pr^ has stronger function compared to NS1^cam^, consistent with the notion that the Puerto Rican isolate is more pathogenic than the Cambodian isolate.

The E protein from the Puerto Rican strain (prM::E^pr^) carries a p.S139N mutation compared to the E protein from the Cambodian strain (prM::E^cam^). This mutation has been associated with increased infectivity and severity of microcephaly when tested in mice and human cells (Yuan et al., 2017). prM::E^pr^ caused semi-lethality when expressed with *Tub-GAL4* (Figs 1C and 7) and exhibited an ERG defect when expressed with *Rh1-GAL4* (Fig. 6). Although we did not observe a difference between prM::E^pr^ and prM::E^cam^ when these proteins were expressed using *Tub-GAL4*, we identified functional differences when *pnr-GAL4* and *Rh1-GAL4* were used. In contrast to prM::E^pr^, which did not show any defect when driven with *pnr-GAL4*, prM::E^cam^ expression using this driver caused lethality (Fig. 7A). Similarly, expression of prM::E^cam^ caused a stronger ERG phenotype compared to that of prM::E^pr^ using *Rh1-GAL4* (Fig. 7B-G). These data indicate that the prM::E^cam^ is more deleterious compared to prM::E^pr^, which was opposite from what we expected. Regardless, these data show that the *Drosophila* assay system can be used to identify functional differences of mutant viral proteins.

## DISCUSSSION

We developed new transgenic *Drosophila* strains that will allow researchers to investigate how viral proteins affect host pathways to cause disease. We expressed all ten ZIKV proteins (some as fused proteins) in eight contexts using three ubiquitous and five tissue-specific GAL4 drivers. With the exception of Capsid, we were able to identify scorable phenotypes for all viral proteins, either when expressed alone or when co-expressed with their known functional partners. These phenotypes were not uniform; each viral protein showed different phenotypes in diverse contexts, suggesting that each protein interferes with specific developmental and cellular processes in distinct tissues. Taken together with published literature on mechanisms of CZS, our work supports a model in which CZS can be driven by multiple molecular mechanisms that are not mutually exclusive. The confluence of these mechanisms may drive disease severity.

ZIKV infection has been linked to severe microcephaly (de Araujo et al., 2018), a devastating phenotype leaving the patient dependent on others throughout their life. Previous reports using a comparative proteomics approach and a *Drosophila* model of microcephaly demonstrated that NS4A expression affects brain development by inhibiting the ANKLE2 pathway (Link et al., 2019; Shah et al., 2018). The results presented here verify this observation and emphasize the reliability of genetic model systems such as the fruit fly. By testing additional ZIKV proteins in the same microcephaly model, we demonstrated that NS2B::NS3 and NS4B also likely contribute to this phenotype. Host pathways disrupted by these proteins may unravel additional mechanisms by which ZIKV causes microcephaly. Furthermore, the established ZIKV-human proteomic interaction data set (Shah et al., 2018) will be valuable in illuminating important host targets of these proteins to provide mechanistic insights into ZIKV-associated diseases for therapeutic development.

During ZIKV infection, each viral protein is expressed at high levels, which may interfere with multiple host pathways simultaneously. Combinatorial expression of multiple ZIKV proteins may also provide insight into how hijacking of multiple developmental pathways coalesces to result in the diversity of established patient outcomes. Given that NS2B::NS3, NS4A and NS4B all give rise to developmental phenotypes in the brain, co-expression of these proteins may result with more severe phenotypes that warrant future exploration. We also noted that the function of NS4A and NS4B can be significantly altered by the presence or absence of the small peptide 2K. Cleavage at the 2K site in different flaviviruses has been linked to membrane rearrangements induced during flavivirus replication (Miller et al., 2007; Roosendaal et al., 2006) or changes in flavivirus protein function (Płaszczyca et al., 2019). Interestingly, the subcellular localization of NS4A::2K and 2K::NS4B differed from that of NS4A and NS4B alone, respectively. It is possible that 2K cleavage is an important regulatory step during viral replication and phenotype development, possibly modulating the function of these proteins or which host proteins each ZIKV protein targets. Alternatively, the small peptide itself could play a role in phenotype development, and expression of the 2K peptide alone could be an interesting path to explore its function.

Comparative analysis of proteins from the Puerto Rican or Cambodian ZIKV strains gave interesting insights into ways in which *Drosophila* can be used to study viral evolution. Modern ZIKV infection is associated with fetal microcephaly; however, earlier strains of the virus presented have not been associated with brain developmental defects (Chu et al., 2017; Duong et al., 2017; Kuadkitkan et al., 2020). Our experiments in which we expressed both the Puerto Rican and Cambodian strains suggest that NS1^pr^ may have more severe effects on the host. However, prM::E^pr^ expression resulted in milder phenotypes than those for prM::E^cam^ expression, as per experiments performed using two GAL4 drivers (*Rh1-GAL4* and *pnr-GAL4*) with two distinct phenotypes (ERG and lethality). This was unexpected as the p.S139N variant present in prM::E^pr^ has been reported to worsen the infection outcome in mice (Yuan et al., 2017). These discrepancies in data may represent a difference in viral effects and mechanisms of action in different tissues. Nevertheless, considering that we observed a phenotypic difference in both cases examined, *Drosophila* could be used as a primary screening system to identify mutations that have a functional consequence and that can be further tested in cellular models or rodent systems.

Finally, our results show that *Drosophila* can be used as a system to explore the post-developmental effects of viral proteins on neuronal function. The *Drosophila* eye is a well-established model to investigate neural function and degenerative phenotypes that are often associated with disease (Ugur et al., 2016). Although expression of ZIKV NS4A caused no obvious phenotypes in young flies, aged animals showed significant reduction in ERG transient amplitudes. Our data suggest that neurons expressing NS4A are functional early in life but may become less functional or degenerate over time. These results implicate that late-onset neuronal dysfunction might be a concerning phenotype following ZIKV infection. Indeed, studies in mouse and human iPSC-based studies also found that exposure to ZIKV can result in neurodegeneration or make neurons more susceptible to insults (Bellmann et al., 2019; Noguchi et al., 2020). Hence, it will be imperative to follow how neuronal function changes after ZIKV infection in children and adults.

## MATERIALS AND METHODS

### *Drosophila melanogaster* strains and culture

The following fly lines were used: *PBac{y[+mDint2] w[+mC]=UAS-Capsid^pr^.HA}VK00037* (this study), *PBac{y[+mDint2] w[+mC]=UAS-prM::E^pr^.HA}VK00037* (this study), *PBac{y[+mDint2] w[+mC]=UAS-prM::E^cam^.HA}VK00037* (this study), *PBac{y[+mDint2] w[+mC]=UAS-NS1^pr^.HA}VK00037* (this study), *PBac{y[+mDint2] w[+mC]=UAS-NS1^cam^.HA}VK00037* (this study), *PBac{y[+mDint2] w[+mC]=UAS-NS2A^pr^.HA}VK00037* (this study), *PBac{y[+mDint2] w[+mC]=UAS-NS2B::NS3^pr^.HA}VK00037* (this study), *PBac{y[+mDint2] w[+mC]= UAS-NS4A^pr^.HA}VK00037* (this study), *PBac{y[+mDint2] w[+mC]= UAS-NS4A::2K^pr^.HA}VK00037* (this study), *PBac{y[+mDint2] w[+mC] =UAS-2K::NS4B^pr^.HA}VK00037* (this study), *PBac{y[+mDint2] w[+mC] =UAS-NS4B^pr^.HA}VK00037* (this study), *PBac{y[+mDint2] w[+mC] =UAS-NS5^pr^.HA}VK00037* (this study), *P{UASt-CD8-GFP}* (Lee and Luo, 2001), *Act-GAL4* (*P{Act5C-GAL4}17bFO1*) (Ito et al., 1997), *insc-GAL4* (*P{w[+mW.hs]=GawB}insc[Mz1407]*) (Luo et al., 1994), *Tub-GAL4 P{w[+mC]=tubP-GAL4}LL7* [*α-Tubulin at 84B* (*αTub84B*)] (Lee and Luo, 1999), *pnr-GAL4 P{w[+mW.hs]=GawB}pnr[MD237]* (RRID: BDSC_3039), *GMR-GAL4 (P{w[+mC]=GAL4-ninaE.GMR}12)* (RRID: BDSC_1104) and *da-GAL4* (*P{w[+mW.hs]=GAL4-da.G32}UH1*) (Wodarz et al., 1995). All flies were maintained at room temperature (∼22°C) and grown on standard cornmeal and molasses medium in plastic vials. Crosses were performed at the temperatures indicated (25°C or 29°C). All studies contained male and female flies. Each cohort was repeated at least three times, sometimes more. Each cohort had multiple crosses. Brain volume measurements were conducted in late third instar larvae (judged by gut clearance and extruding spiracles) and all other assessments occurred in adults. All novel *Drosophila* lines were deposited to the Bloomington *Drosophila* Stock Center.

### Generation of transgenic constructs

To generate ZIKV expression constructs, the ZIKV sequence was acquired from the strain PRVABC-59 (GenBank: KX377337.1), an Asian lineage isolated from Puerto Rico in 2015. Constructs were designed as described in Shah et al. (2018). All sequences were codon optimized for expression in *Drosophila* using the Codon Optimization Tool (Integrated DNA Technologies; https://www.idtdna.com/CodonOpt). For initial fragments (Capsid, prM::E, NS1, NS2A, NS2B::NS3, NS4A, 2K::NS4B and NS5), double-stranded, linear gene fragments were generated using gBlocks (Integrated DNA Technologies). ZIKV fragments were synthesized with additional regions of homology that correspond to sequences in the destination vector (pGW-HA.attB, a gift from Konrad Basler, Institute of Molecular Life Sciences, University of Zurich; Bischof et al., 2013) at the 5′ (5′-TCAAAAGGTATAGGAACTTCAACCGGTCAAC-3′, Kozak sequence underlined) and 3′ (5′-GTACCTCGAAGTTCCTATTCTCTACTTAGTATA-3′) ends. AgeI and KpnI restriction enzymes were used to remove the gateway compatible cassette from pGW-HA.attB, removing the *attR1*, *CmR*, *ccdB* and *attR2* regions. UAS expression constructs were generated by combining the linearized pGW-HA.attB backbone and each individual ZIKV fragment in Gibson assembly reactions (Gibson Assembly Cloning Kit, E5510S, New England Biolabs). To generate the NS4A::2K construct, the NS4A and 2K peptide constructs (with 25 bp overlapping regions included in primers) were PCR amplified, gel purified and used in a three-fragment Gibson assembly reaction. To generate the NS4B construct without the 2K peptide, the NS4B construct was PCR amplified, gel purified and assembled with pGW-HA.attB using Gibson assembly. To generate prM::E and NS1 constructs containing single amino acid changes seen in the Cambodian strain (FSS13025), variant products were synthesized and cloned into pGW-HA.attB using Gibson assembly as above. All constructs were inserted into the VK37 landing site on the second chromosome in *D. melanogaster* by microinjection into a strain expressing ɸC31 integrase in the germline (y[1] M{RFP[3xP3.PB] GFP[E.3xP3]=vas-int.Dm}ZH-2A w[*]; PBac{y[+]-attP-3B}VK00037) (Bischof et al., 2007; Venken et al., 2006). Transgenic lines were selected by the *white^+^* marker in a *white^−^* background, and stable stocks were established through standard procedures (Harnish et al., 2019).

### Crossing schemes and scoring of gross phenotypes

Wing, eye and thorax phenotypes were explored by driving the *UAS-ZIKV* cDNA lines with *nub-GAL4*, *GMR-GAL4 and pnr-GAL4*, respectively. Virgin females from the GAL4 stocks were collected from stock bottles kept at 25°C. Male flies were collected from the UAS stocks. Virgin females were isolated for 24 h before being crossed, and three to four females with three to five males were placed in the same vial containing standard cornmeal and molasses medium. Crosses were initially maintained at 25°C. The crosses were transferred into new vials after 4-6 days and maintained at 25°C. After 4-6 days, these parental flies were again transferred to new vials and maintained at 29°C. New flies were added if there was an insufficient number for the cross at 29°C. Flies were collected 6-8 days after flies had begun to eclose. An example of the number of flies assessed for each cross is given in Table S3. F1 progeny were scored for gross morphological phenotypes after being anesthetized with carbon dioxide. If phenotypes were observed, the flies were sacrificed via overnight freezing at −20°C and imaged the following day. The wings were dissected dry in a siliconized dish and imaged at 5× magnification. To assess lethality, the rate of flies carrying balancer chromosomes in the F1 generation was compared to the expected rate to determine whether flies were eclosing according to Mendelian ratios. Semi-lethality was noted when the observed number of protein-expressing flies was less than 75% of the expected number based on Mendelian ratios.

### Immunostaining of larval brains

Late third instar (judged based on gut clearance and extruding spiracles) larval brains were dissected in PBS and fixed with 4% paraformaldehyde in PBS containing 0.3% Triton X-100 for 20 min. For immunostaining, brains were blocked in PBS containing 0.3% Triton X-100, 1% bovine serum albumin (BSA) and 5% normal goat serum and incubated in primary antibody in PBS containing 0.3% Triton X-100 and 1% BSA overnight. The primary antibodies included rat anti-Deadpan (Bier et al., 1992) (Abcam, ab195172, 1:250 or 1:500), mouse anti-Prospero (Spana and Doe, 1995) (Developmental Studies Hybridoma Bank, MR1A, 1:1000), mouse anti-Cnx99A (Riedel et al., 2016) (Developmental Studies Hybridoma Bank, Cnx99A 6-2-1, 1:100), rabbit anti-phospho-histone H3 (Ser10) (Millipore Sigma, 06-570, 1:1000), rabbit anti-cleaved *Drosophila* Dcp1 (Asp215) (Cell Signaling Technology, 9578, 1:100 to 1:500) and mouse anti-HA (Covance, 901501, 1:1000) with donkey secondary antibodies from Jackson ImmunoResearch (712-605-153, 715-295-151 and 711-295-152) used at 1:500. Brains were mounted with double-sided tape spacers and imaged using a confocal microscope (Leica SP8) with 2 or 3 µm sections through the entire brain lobe.

### Brain volume measurement

Brains from third instar larvae were stained and mounted with tape spacers and imaged using a Leica SP8 microscope with 2 or 3 µm sections through the entire brain lobe. The resulting stacks were analyzed using the Surfaces function in Imaris (Bitplane) to quantify brain lobe volume (in μm^3^). One lobe from each brain was imaged and a total of ten brains was analyzed per genotype or condition. Brain lobe volumes are displayed as individual values with the line representing the median. Statistical significance was determined using one-way ANOVA with multiple comparisons posttest calculated using GraphPad Prism. Brain volumes are displayed as total brain volume (μm^3^). A post hoc power analysis was conducted using RStudio (2023.09.01+494). The average of all treatment averages was used to calculate the effect size. *n*=7.8 animals needed for 80% confidence at *P*=0.05.

More than five Dpn^+^ central brain neuroblasts were assessed and imaged for high-quality images for subcellular localization. However, numerous lower magnification images were acquired and assessed (every brain lobe for volume, cell division and cell death; *n*>20)

### ERG recordings

*Rh1*-*GAL4* was crossed to each ZIKV expression line, progeny were collected and aged at 25°C with a 12 h/12 h light/dark cycle (500 lux) for 30 days. ERG recordings were performed as follows: flies were immobilized on glass slides with Elmer's glue, a glass reference electrode filed with 100 mM NaCl was inserted behind the eye and a sharp recording electrode filled with 100 mM NaCl was placed on the eye. Light flashes of 1 s followed by 1 s recovery were delivered using a halogen lamp. Eight to ten flies were tested for each genotype. Data were recorded and analyzed using AxoScope pClamp (Molecular Devices).

### Transmission electron microscopy

Retinas were imaged following standard electron microscopy procedures. Whole heads were dissected and fixed in 2% paraformaldehyde and 2.5% glutaraldehyde in 0.1 M sodium cacodylate buffer (pH 7.2) overnight on a rotator, rinsed with water three times, and post fixed with 1% aqueous osmium tetroxide. Samples were rinsed again three times with water and moved through an ethanol dehydration series (30-100%) with propylene oxide as the final wash. Samples were gradually permeated with three ratios of propylene oxide and Embed 812 (Electron Microscopy Sciences) and finally three changes of pure resin overnight on a rotator. Retinas were embedded in flat silicone molds and cured at 62°C in an oven for 3 days. Samples were then sectioned, stained with 1% uranyl acetate for 10 min, stained with lead citrate for 1 min, then examined by transmission electron microscopy using a JEOL JEM 1010 transmission electron microscope (JEOL USA) at 80 kV. An AMT XR16 CCD camera with exposure of 1100 ms, gain 2.7, bin 1 and sigma 1.65 was used.

## Supplementary Material

10.1242/dmm.050297_sup1 Supplementary information

## References

[DMM050297C1] Agnès, F., Suzanne, M. and Noselli, S. (1999). The Drosophila JNK pathway controls the morphogenesis of imaginal discs during metamorphosis. *Development.* 126, 5453-5462. 10.1242/dev.126.23.5453 10556069

[DMM050297C2] Aragao, M., Holanda, A. C., Brainer-Lima, A. M., Petribu, N. C. L., Castillo, M., van der Linden, V., Serpa, S. C., Tenorio, A. G., Travassos, P. T. C., Cordeiro, M. T. et al. (2017). Nonmicrocephalic infants with congenital Zika syndrome suspected only after neuroimaging evaluation compared with those with microcephaly at birth and postnatally: how large is the Zika Virus “Iceberg”? *AJNR Am. J. Neuroradiol.* 38, 1427-1434. 10.3174/ajnr.A5216 28522665 PMC7959892

[DMM050297C3] Asadi-Pooya, A. A. (2016). Zika virus-associated seizures. *Seizure* 43, 13. 10.1016/j.seizure.2016.10.011 27788398

[DMM050297C4] Bellmann, J., Monette, A., Tripathy, V., Sójka, A., Abo-Rady, M., Janosh, A., Bhatnagar, R., Bickle, M., Mouland, A. J. and Sterneckert, J. (2019). Viral Infections Exacerbate FUS-ALS Phenotypes in iPSC-Derived Spinal Neurons in a Virus Species-Specific Manner. *Front Cell Neurosci.* 13, 480. 10.3389/fncel.2019.00480 31695598 PMC6817715

[DMM050297C5] Bertolli, J., Attell, J. E., Rose, C., Moore, C. A., Melo, F., Staples, J. E., Kotzky, K., Krishna, N., Satterfield-Nash, A., Pereira, I. O. et al. (2020). Functional outcomes among a cohort of children in northeastern brazil meeting criteria for follow-up of congenital Zika virus infection. *Am. J. Trop. Med. Hyg.* 102, 955-963. 10.4269/ajtmh.19-0961 32228785 PMC7204564

[DMM050297C6] Bier, E. (2005). Drosophila, the golden bug, emerges as a tool for human genetics. *Nat. Rev. Genet.* 6, 9-23. 10.1038/nrg1503 15630418

[DMM050297C7] Bier, E., Vaessin, H., Younger-Shepherd, S., Jan, L. Y. and Jan, Y. N. (1992). deadpan, an essential pan-neural gene in Drosophila, encodes a helix-loop-helix protein similar to the hairy gene product. *Genes Dev.* 6, 2137-2151. 10.1101/gad.6.11.2137 1427077

[DMM050297C8] Bischof, J., Maeda, R. K., Hediger, M., Karch, F. and Basler, K. (2007). An optimized transgenesis system for Drosophila using germ-line-specific phiC31 integrases. *Proc. Natl. Acad. Sci. USA* 104, 3312-3317. 10.1073/pnas.0611511104 17360644 PMC1805588

[DMM050297C9] Bischof, J., Björklund, M., Furger, E., Schertel, C., Taipale, J. and Basler, K. (2013). A versatile platform for creating a comprehensive UAS-ORFeome library in Drosophila. *Development* 140, 2434-2442. 10.1242/dev.088757 23637332

[DMM050297C10] Brand, A. H. and Perrimon, N. (1993). Targeted gene expression as a means of altering cell fates and generating dominant phenotypes. *Development* 118, 401-415. 10.1242/dev.118.2.401 8223268

[DMM050297C11] Cao-Lormeau, V. M., Blake, A., Mons, S., Lastere, S., Roche, C., Vanhomwegen, J., Dub, T., Baudouin, L., Teissier, A., Larre, P. et al. (2016). Guillain-Barre Syndrome outbreak associated with Zika virus infection in French Polynesia: a case-control study. *Lancet* 387, 1531-1539. 10.1016/S0140-6736(16)00562-6 26948433 PMC5444521

[DMM050297C12] Carteaux, G., Maquart, M., Bedet, A., Contou, D., Brugieres, P., Fourati, S., Cleret de Langavant, L., de Broucker, T., Brun-Buisson, C., Leparc-Goffart, I. et al. (2016). Zika virus associated with meningoencephalitis. *N. Engl. J. Med.* 374, 1595-1596. 10.1056/NEJMc1602964 26958738

[DMM050297C13] Carvalho, A., Brites, C., Mochida, G., Ventura, P., Fernandes, A., Lage, M. L., Taguchi, T., Brandi, I., Silva, A., Franceschi, G. et al. (2019). Clinical and neurodevelopmental features in children with cerebral palsy and probable congenital Zika. *Brain Dev.* 41, 587-594. 10.1016/j.braindev.2019.03.005 30914212

[DMM050297C14] Cauchemez, S., Besnard, M., Bompard, P., Dub, T., Guillemette-Artur, P., Eyrolle-Guignot, D., Salje, H., Van Kerkhove, M. D., Abadie, V., Garel, C. et al. (2016). Association between Zika virus and microcephaly in French Polynesia, 2013-15: a retrospective study. *Lancet* 387, 2125-2132. 10.1016/S0140-6736(16)00651-6 26993883 PMC4909533

[DMM050297C15] Chu, V., Petersen, L., Moore, C., Meaney-Delman, D., Nelson, G., Sonne, D. C., Glaser, C. and Rasmussen, S. (2017). Congenital Zika Syndrome (CZS) phenotype seen in older children. *Open Forum Infectious Diseases* 4, S696-S697. 10.1093/ofid/ofx163.1867

[DMM050297C16] Conde, J. N., Schutt, W. R., Mladinich, M., Sohn, S.-Y., Hearing, P. and Mackow, E. R. (2020). NS5 sumoylation directs nuclear responses that permit Zika virus to persistently infect human brain microvascular endothelial cells. *J. Virol.* 94, e01086-e01020. 10.1128/JVI.01086-20 32699085 PMC7495392

[DMM050297C17] Cortese, M., Goellner, S., Acosta, E. G., Neufeldt, C. J., Oleksiuk, O., Lampe, M., Haselmann, U., Funaya, C., Schieber, N., Ronchi, P. et al. (2017). Ultrastructural Characterization of Zika Virus Replication Factories. *Cell Rep.* 18, 2113-2123. 10.1016/j.celrep.2017.02.014 28249158 PMC5340982

[DMM050297C18] da Silva Pone, M. V., Moura Pone, S., Araujo Zin, A., Barros Mendes, P. H., Senra Aibe, M., Barroso de Aguiar, E. and de Oliveira Gomes da Silva, T. (2018). Zika virus infection in children: epidemiology and clinical manifestations. *Childs Nerv. Syst.* 34, 63-71. 10.1007/s00381-017-3635-3 29110197

[DMM050297C19] de Araujo, T. V. B., Ximenes, R. A. A., Miranda-Filho, D. B., Souza, W. V., Montarroyos, U. R., de Melo, A. P. L., Valongueiro, S., de Albuquerque, M., Braga, C., Filho, S. P. B. et al. (2018). Association between microcephaly, Zika virus infection, and other risk factors in Brazil: final report of a case-control study. *Lancet Infect. Dis.* 18, 328-336. 10.1016/S1473-3099(17)30727-2 29242091 PMC7617036

[DMM050297C20] Deal, S. L. and Yamamoto, S. (2018). Unraveling novel mechanisms of neurodegeneration through a large-scale forward genetic screen in Drosophila. *Front. Genet* 9, 700. 10.3389/fgene.2018.00700 30693015 PMC6339878

[DMM050297C21] Delatorre, E., Mir, D. and Bello, G. (2017). Tracing the origin of the NS1 A188V substitution responsible for recent enhancement of Zika virus Asian genotype infectivity. *Mem. Inst. Oswaldo Cruz* 112, 793-795. 10.1590/0074-02760170299 28876359 PMC5661894

[DMM050297C22] Dolph, P., Nair, A. and Raghu, P. (2011). Electroretinogram recordings of Drosophila. *Cold Spring Harb. Protoc.* 2011, pdb prot5549. 10.1101/pdb.prot5549 21205849

[DMM050297C23] Duong, V., Ong, S., Leang, R., Huy, R., Ly, S., Mounier, U., Ou, T., In, S., Peng, B., Ken, S. et al. (2017). Low circulation of Zika virus, cambodia, 2007-2016. *Emerg. Infect. Dis.* 23, 296-299. 10.3201/eid2302.161432 27875110 PMC5324809

[DMM050297C24] Falgout, B., Pethel, M., Zhang, Y. M. and Lai, C. J. (1991). Both nonstructural proteins NS2B and NS3 are required for the proteolytic processing of dengue virus nonstructural proteins. *J. Virol.* 65, 2467-2475. 10.1128/JVI.65.5.2467-2475.1991 2016768 PMC240601

[DMM050297C25] Fishburn, A. T., Pham, O. H., Kenaston, M. W., Beesabathuni, N. S. and Shah, P. S. (2022). Let's get physical: flavivirus-host protein-protein interactions in replication and pathogenesis. *Front. Microbiol.* 13, 847588. 10.3389/fmicb.2022.847588 35308381 PMC8928165

[DMM050297C26] Gibson, D. G., Young, L., Chuang, R.-Y., Venter, J. C., Hutchison, C. A. and Smith, H. O. (2009). Enzymatic assembly of DNA molecules up to several hundred kilobases. *Nat. Methods* 6, 343-345. 10.1038/nmeth.1318 19363495

[DMM050297C27] Harnish, J. M., Deal, S. L., Chao, H.-T., Wangler, M. F. and Yamamoto, S. (2019). In vivo functional study of disease-associated rare human variants using Drosophila. *J. Vis. Exp*. 150, e59658. 10.3791/59658 PMC7418855 31498321

[DMM050297C28] Harnish, J. M., Link, N. and Yamamoto, S. (2021). Drosophila as a model for infectious diseases. *Int. J. Mol. Sci.* 22, 2724. 10.3390/ijms22052724 33800390 PMC7962867

[DMM050297C29] Hou, W., Cruz-Cosme, R., Armstrong, N., Obwolo, L. A., Wen, F., Hu, W., Luo, M.-H. and Tang, Q. (2017). Molecular cloning and characterization of the genes encoding the proteins of Zika virus. *Gene.* 628, 117-128. 10.1016/j.gene.2017.07.049 28720531 PMC5729740

[DMM050297C30] Ito, K., Awano, W., Suzuki, K., Hiromi, Y. and Yamamoto, D. (1997). The Drosophila mushroom body is a quadruple structure of clonal units each of which contains a virtually identical set of neurones and glial cells. *Development* 124, 761-771. 10.1242/dev.124.4.761 9043058

[DMM050297C31] Kesari, A. S., Heintz, V. J., Poudyal, S., Miller, A. S., Kuhn, R. J. and LaCount, D. J. (2020). Zika virus NS5 localizes at centrosomes during cell division. *Virology* 541, 52-62. 10.1016/j.virol.2019.11.018 32056715 PMC7216507

[DMM050297C32] Khan, M. A., Windpassinger, C., Ali, M. Z., Zubair, M., Gul, H., Abbas, S., Khan, S., Badar, M., Mohammad, R. M. and Nawaz, Z. (2017). Molecular genetic analysis of consanguineous families with primary microcephaly identified pathogenic variants in the ASPM gene. *J. Genet.* 96, 383-387. 10.1007/s12041-017-0759-x 28674240

[DMM050297C33] Kuadkitkan, A., Wikan, N., Sornjai, W. and Smith, D. R. (2020). Zika virus and microcephaly in Southeast Asia: A cause for concern? *J Infect Public Health* 13, 11-15. 10.1016/j.jiph.2019.09.012 31669035

[DMM050297C34] Kumar, J. P. (2001). Signalling pathways in Drosophila and vertebrate retinal development. *Nat. Rev. Genet.* 2, 846-857. 10.1038/35098564 11715040

[DMM050297C35] Lanciotti, R. S., Lambert, A. J., Holodniy, M., Saavedra, S. and Signor Ldel, C. (2016). Phylogeny of Zika virus in western hemisphere, 2015. *Emerg. Infect. Dis.* 22, 933-935. 10.3201/eid2205.160065 27088323 PMC4861537

[DMM050297C72] Lee, T. and Luo, L. (1999). Mosaic analysis with a repressible cell marker for studies of gene function in neuronal morphogenesis. *Neuron* 22, 451-461. 10.1016/s0896-6273(00)80701-1 10197526

[DMM050297C73] Lee, T. and Luo, L. (2001). Mosaic analysis with a repressible cell marker (MARCM) for Drosophila neural development. *Trends Neurosci.* 24, 251-254. 10.1016/s0166-2236(00)01791-4 11311363

[DMM050297C36] Li, P., Wu, J., Liu, S., Lu, R., Jiang, H., Wang, N., Luo, M., Guo, L., Xiao, J., Bu, L. et al. (2022). The RNA polymerase of cytoplasmically replicating Zika virus binds with chromatin DNA in nuclei and regulates host gene transcription. *Proc. Natl. Acad. Sci. USA* 119, e2205013119. 10.1073/pnas.2205013119 36442102 PMC9894162

[DMM050297C37] Liang, Q., Luo, Z., Zeng, J., Chen, W., Foo, S. S., Lee, S. A., Ge, J., Wang, S., Goldman, S. A., Zlokovic, B. V. et al. (2016). Zika Virus NS4A and NS4B Proteins Deregulate Akt-mTOR Signaling in Human Fetal Neural Stem Cells to Inhibit Neurogenesis and Induce Autophagy. *Cell Stem Cell* 19, 663-671. 10.1016/j.stem.2016.07.019 27524440 PMC5144538

[DMM050297C38] Lin, C., Amberg, S. M., Chambers, T. J. and Rice, C. M. (1993). Cleavage at a novel site in the NS4A region by the yellow fever virus NS2B-3 proteinase is a prerequisite for processing at the downstream 4A/4B signalase site. *J. Virol.* 67, 2327-2335. 10.1128/JVI.67.4.2327-2335.1993 8445732 PMC240389

[DMM050297C39] Link, N., Chung, H., Jolly, A., Withers, M., Tepe, B., Arenkiel, B. R., Shah, P. S., Krogan, N. J., Aydin, H., Geckinli, B. B. et al. (2019). Mutations in ANKLE2, a Zika Virus target, disrupt an asymmetric cell division pathway in drosophila neuroblasts to cause microcephaly. *Dev. Cell* 51, 713-729.e6. 10.1016/j.devcel.2019.10.009 31735666 PMC6917859

[DMM050297C40] Liu, Y., Liu, J., Du, S., Shan, C., Nie, K., Zhang, R., Li, X.-F., Zhang, R., Wang, T., Qin, C.-F. et al. (2017). Evolutionary enhancement of Zika virus infectivity in Aedes aegypti mosquitoes. *Nature* 545, 482-486. 10.1038/nature22365 28514450 PMC5885636

[DMM050297C41] Luo, L., Liao, Y. J., Jan, L. Y. and Jan, Y. N. (1994). Distinct morphogenetic functions of similar small GTPases: Drosophila Drac1 is involved in axonal outgrowth and myoblast fusion. *Genes Dev.* 8, 1787-1802. 10.1101/gad.8.15.1787 7958857

[DMM050297C42] Martin-Blanco, E., Pastor-Pareja, J. C. and Garcia-Bellido, A. (2000). JNK and decapentaplegic signaling control adhesiveness and cytoskeleton dynamics during thorax closure in Drosophila. *Proc. Natl. Acad. Sci. USA* 97, 7888-7893. 10.1073/pnas.97.14.7888 10884420 PMC16640

[DMM050297C43] Medina, M. T. and Medina-Montoya, M. (2017). New spectrum of the neurologic consequences of Zika. *J. Neurol. Sci.* 383, 214-215. 10.1016/j.jns.2017.10.046 29108750

[DMM050297C44] Medina, M. T., England, J. D., Lorenzana, I., Medina-Montoya, M., Alvarado, D., De Bastos, M., Fontiveros, S., Sierra, M. and Contreras, F. (2016). Zika virus associated with sensory polyneuropathy. *J. Neurol. Sci.* 369, 271-272. 10.1016/j.jns.2016.08.044 27653905

[DMM050297C45] Mehta, R., Soares, C. N., Medialdea-Carrera, R., Ellul, M., da Silva, M. T. T., Rosala-Hallas, A., Jardim, M. R., Burnside, G., Pamplona, L., Bhojak, M. et al. (2018). The spectrum of neurological disease associated with Zika and chikungunya viruses in adults in Rio de Janeiro, Brazil: a case series. *PLoS Negl. Trop. Dis.* 12, e0006212. 10.1371/journal.pntd.0006212 29432457 PMC5837186

[DMM050297C46] Miller, S., Kastner, S., Krijnse-Locker, J., Buhler, S. and Bartenschlager, R. (2007). The non-structural protein 4A of dengue virus is an integral membrane protein inducing membrane alterations in a 2K-regulated manner. *J. Biol. Chem.* 282, 8873-8882. 10.1074/jbc.M609919200 17276984

[DMM050297C47] Nem de Oliveira Souza, I., Frost, P. S., Franca, J. V., Nascimento-Viana, J. B., Neris, R. L. S., Freitas, L., Pinheiro, D., Nogueira, C. O., Neves, G., Chimelli, L. et al. (2018). Acute and chronic neurological consequences of early-life Zika virus infection in mice. *Sci Transl Med.* 10, eaar2749. 10.1126/scitranslmed.aar2749 29875203

[DMM050297C48] Noguchi, K. K., Swiney, B. S., Williams, S. L., Huffman, J. N., Lucas, K., Wang, S. H., Kapral, K. M., Li, A. and Dikranian, K. T. (2020). Zika virus infection in the developing mouse produces dramatically different neuropathology dependent on viral strain. *J. Neurosci.* 40, 1145-1161. 10.1523/JNEUROSCI.1376-19.2019 31836659 PMC6988996

[DMM050297C49] Petit, M. J., Kenaston, M. W., Pham, O. H., Nagainis, A. A., Fishburn, A. T. and Shah, P. S. (2021). Nuclear dengue virus NS5 antagonizes expression of PAF1-dependent immune response genes. *PLoS Pathog*. 17, e1010100. 10.1371/journal.ppat.1010100 34797876 PMC8641875

[DMM050297C74] Płaszczyca, A., Scaturro, P., Neufeldt, C. J., Cortese, M., Cerikan, B., Ferla, S., Brancale, A., Pichlmair, A. and Bartenschlager, R. (2019). A novel interaction between dengue virus nonstructural protein 1 and the NS4A-2K-4B precursor is required for viral RNA replication but not for formation of the membranous replication organelle. *PLoS Pathog*. 15, e1007736. 10.1371/journal.ppat.1007736 31071189 PMC6508626

[DMM050297C50] Raper, J., Kovacs-Balint, Z., Mavigner, M., Gumber, S., Burke, M. W., Habib, J., Mattingly, C., Fair, D., Earl, E., Feczko, E. et al. (2020). Long-term alterations in brain and behavior after postnatal Zika virus infection in infant macaques. *Nat. Commun.* 11, 2534. 10.1038/s41467-020-16320-7 32439858 PMC7242369

[DMM050297C51] Rice, M. E., Galang, R. R., Roth, N. M., Ellington, S. R., Moore, C. A., Valencia-Prado, M., Ellis, E. M., Tufa, A. J., Taulung, L. A., Alfred, J. M. et al. (2018). Vital signs: Zika-associated birth defects and neurodevelopmental abnormalities possibly associated with congenital Zika virus infection - U.S. territories and freely associated states, 2018. *MMWR Morb. Mortal. Wkly. Rep.* 67, 858-867. 10.15585/mmwr.mm6731e1 30091967 PMC6089332

[DMM050297C52] Riedel, F., Gillingham, A. K., Rosa-Ferreira, C., Galindo, A. and Munro, S. (2016). An antibody toolkit for the study of membrane traffic in Drosophila melanogaster. *Biol. Open* 5, 987-992. 10.1242/bio.018937 27256406 PMC4958275

[DMM050297C53] Roosendaal, J., Westaway, E. G., Khromykh, A. and Mackenzie, J. M. (2006). Regulated cleavages at the West Nile virus NS4A-2K-NS4B junctions play a major role in rearranging cytoplasmic membranes and Golgi trafficking of the NS4A protein. *J. Virol.* 80, 4623-4632. 10.1128/JVI.80.9.4623-4632.2006 16611922 PMC1472005

[DMM050297C54] Schweisguth, F. (2015). Asymmetric cell division in the Drosophila bristle lineage: from the polarization of sensory organ precursor cells to Notch-mediated binary fate decision. *Wiley Interdiscip Rev. Dev. Biol.* 4, 299-309. 10.1002/wdev.175 25619594 PMC4671255

[DMM050297C55] Schweisguth, F., Gho, M. and Lecourtois, M. (1996). Control of cell fate choices by lateral signaling in the adult peripheral nervous system of Drosophila melanogaster. *Dev. Genet.* 18, 28-39. 10.1002/(SICI)1520-6408(1996)18:1<28::AID-DVG4>3.0.CO;2-3 8742832

[DMM050297C56] Shah, P. S., Link, N., Jang, G. M., Sharp, P. P., Zhu, T., Swaney, D. L., Johnson, J. R., Von Dollen, J., Ramage, H. R., Satkamp, L. et al. (2018). Comparative flavivirus-host protein interaction mapping reveals mechanisms of dengue and Zika virus pathogenesis. *Cell* 175, 1931-1945.e18. 10.1016/j.cell.2018.11.028 30550790 PMC6474419

[DMM050297C57] Spana, E. P. and Doe, C. Q. (1995). The prospero transcription factor is asymmetrically localized to the cell cortex during neuroblast mitosis in *Drosophila*. *Development* 121, 3187-3195. 10.1242/dev.121.10.3187 7588053

[DMM050297C58] Sun, G., Larsen, C. N., Baumgarth, N., Klem, E. B. and Scheuermann, R. H. (2017). Comprehensive annotation of mature peptides and genotypes for Zika virus. *PLoS One* 12, e0170462. 10.1371/journal.pone.0170462 28125631 PMC5268401

[DMM050297C59] Thomas, B. J. and Wassarman, D. A. (1999). A fly's eye view of biology. *Trends Genet.* 15, 184-190. 10.1016/s0168-9525(99)01720-5 10322485

[DMM050297C60] Ugur, B., Chen, K. and Bellen, H. J. (2016). Drosophila tools and assays for the study of human diseases. *Dis Model Mech.* 9, 235-244. 10.1242/dmm.023762 26935102 PMC4833332

[DMM050297C61] van der Linden, V., Pessoa, A., Dobyns, W., Barkovich, A. J., Junior, H. V., Filho, E. L., Ribeiro, E. M., Leal, M. C., Coimbra, P. P., Aragao, M. F. et al. (2016). Description of 13 infants born during october 2015-january 2016 with congenital Zika virus infection without microcephaly at birth - Brazil. *MMWR Morb. Mortal. Wkly. Rep.* 65, 1343-1348. 10.15585/mmwr.mm6547e2 27906905

[DMM050297C62] Venken, K. J., He, Y., Hoskins, R. A. and Bellen, H. J. (2006). P[acman]: a BAC transgenic platform for targeted insertion of large DNA fragments in D. melanogaster. *Science* 314, 1747-1751. 10.1126/science.1134426 17138868

[DMM050297C63] Wang, D., Chen, C., Liu, S., Zhou, H., Yang, K., Zhao, Q., Ji, X., Chen, C., Xie, W., Wang, Z. et al. (2017). A mutation identified in neonatal microcephaly destabilizes Zika virus ns1 assembly in vitro. *Sci. Rep.* 7, 42580. 10.1038/srep42580 28198446 PMC5309781

[DMM050297C64] Wodarz, A., Hinz, U., Engelbert, M. and Knust, E. (1995). Expression of crumbs confers apical character on plasma membrane domains of ectodermal epithelia of Drosophila. *Cell* 82, 67-76. 10.1016/0092-8674(95)90053-5 7606787

[DMM050297C65] Xia, H., Luo, H., Shan, C., Muruato, A. E., Nunes, B. T. D., Medeiros, D. B. A., Zou, J., Xie, X., Giraldo, M. I., Vasconcelos, P. F. C. et al. (2018). An evolutionary NS1 mutation enhances Zika virus evasion of host interferon induction. *Nat. Commun.* 9, 414. 10.1038/s41467-017-02816-2 29379028 PMC5788864

[DMM050297C66] Yamamoto, S., Jaiswal, M., Charng, W. L., Gambin, T., Karaca, E., Mirzaa, G., Wiszniewski, W., Sandoval, H., Haelterman, N. A., Xiong, B. et al. (2014). A drosophila genetic resource of mutants to study mechanisms underlying human genetic diseases. *Cell* 159, 200-214. 10.1016/j.cell.2014.09.002 25259927 PMC4298142

[DMM050297C67] Yoon, K. J., Song, G., Qian, X., Pan, J., Xu, D., Rho, H. S., Kim, N. S., Habela, C., Zheng, L., Jacob, F. et al. (2017). Zika-virus-encoded NS2A disrupts mammalian cortical neurogenesis by degrading adherens junction proteins. *Cell Stem Cell* 21, 349-358.e6. 10.1016/j.stem.2017.07.014 28826723 PMC5600197

[DMM050297C68] Yu, I.-M., Zhang, W., Holdaway, H. A., Li, L., Kostyuchenko, V. A., Chipman, P. R., Kuhn, R. J., Rossmann, M. G. and Chen, J. (2008). Structure of the immature dengue virus at low pH primes proteolytic maturation. *Science* 319, 1834-1837. 10.1126/science.1153264 18369148

[DMM050297C69] Yuan, L., Huang, X. Y., Liu, Z. Y., Zhang, F., Zhu, X. L., Yu, J. Y., Ji, X., Xu, Y. P., Li, G., Li, C. et al. (2017). A single mutation in the prM protein of Zika virus contributes to fetal microcephaly. *Science* 358, 933-936. 10.1126/science.aam7120 28971967

[DMM050297C70] Zeng, J., Dong, S., Luo, Z., Xie, X., Fu, B., Li, P., Liu, C., Yang, X., Chen, Y., Wang, X. et al. (2020). The Zika virus capsid disrupts corticogenesis by suppressing dicer activity and miRNA biogenesis. *Cell Stem Cell* 27, 618-632.e9. 10.1016/j.stem.2020.07.012 32763144 PMC7541724

[DMM050297C71] Zhao, Z., Tao, M., Han, W., Fan, Z., Imran, M., Cao, S. and Ye, J. (2021). Nuclear localization of Zika virus NS5 contributes to suppression of type I interferon production and response. *J. Gen. Virol.* 102, 001376. 10.1099/jgv.0.001376 31859616 PMC8515865

