# Supplementary material for: A Zika virus protein expression screen in Drosophila to investigate targeted host pathways during development
Source: Dis Model Mech. 2024 Feb 28;17(2):dmm050297. doi: 10.1242/dmm.050297 (PMC10924231; doi:10.1242/dmm.050297)
Supplement: Supplementary information [file dmm-17-050297-s1.pdf]

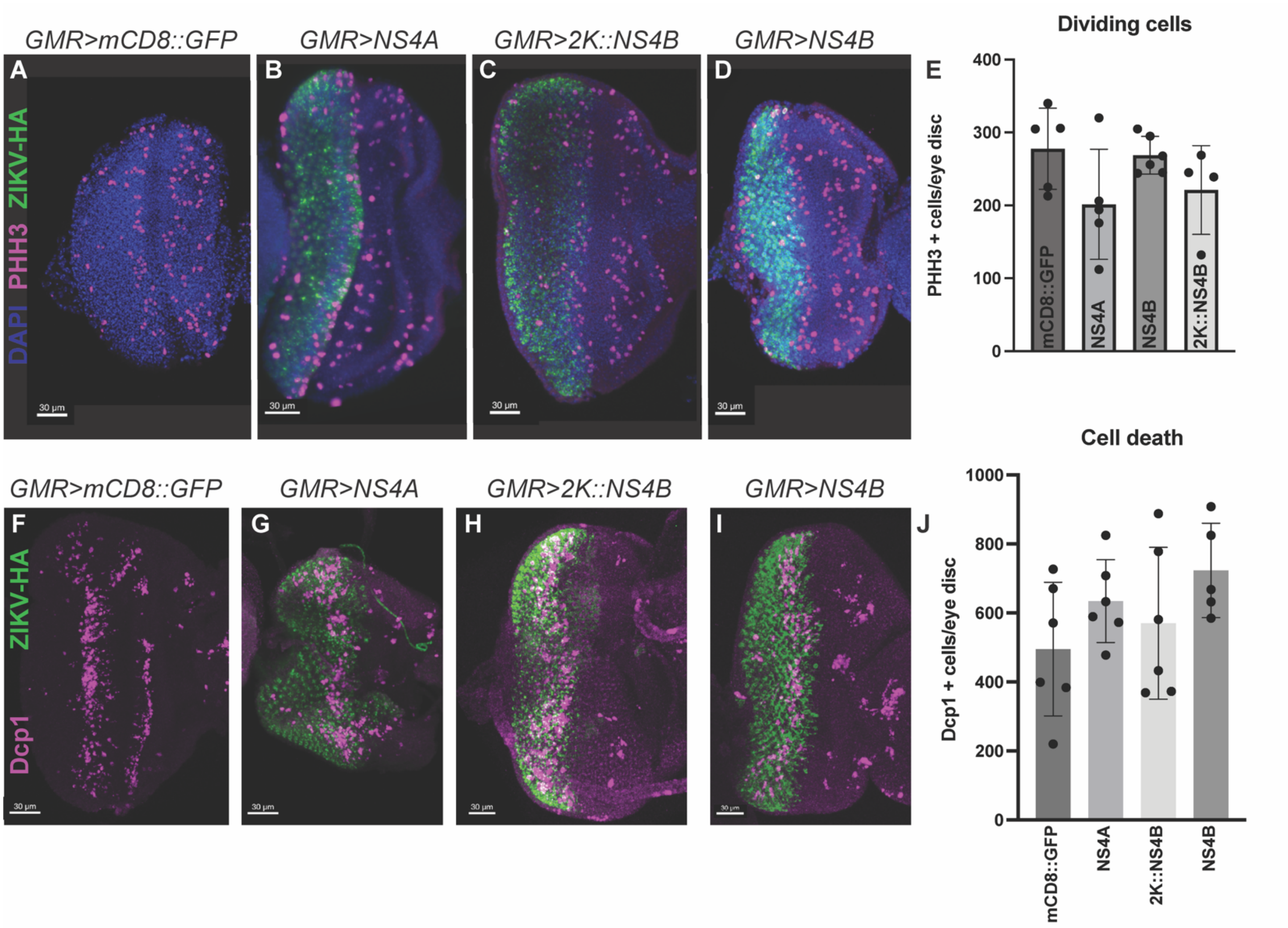

Fig. S1. Expression of NS4A, 2K::NS4B, or NS4B does not overtly affect cell division or cell death in the 3rd instar eye disc. (A-E) 3rd instar eye discs from animals expressing (A) mCD8::GFP (control), (B) NS4A, (C) 2K::NS4B, or (D) NS4B stained for phospho-histone H3 (PHH3, magenta) which marks dividing cells, HA (ZIKV-HA, green), and DAPI (blue). The number of PHH3 positive cells in the eye disc is plotted in (E). No significant changes were found. (F-J) 3rd instar eye discs from animals expressing (A) mCD8::GFP (control), (B) NS4A, (C) 2K::NS4B, or (D) NS4B stained for activated Dcp1 (magenta) which indicates active *Drosophila* caspase 1 and is an indicator of apoptosis, and HA (ZIKV-HA, green). No significant differences were found, but a trend of increased cell death was noted. One way ANOVA with multi-comparisons posttest.

Disease Models & Mechanisms • Supplementary information

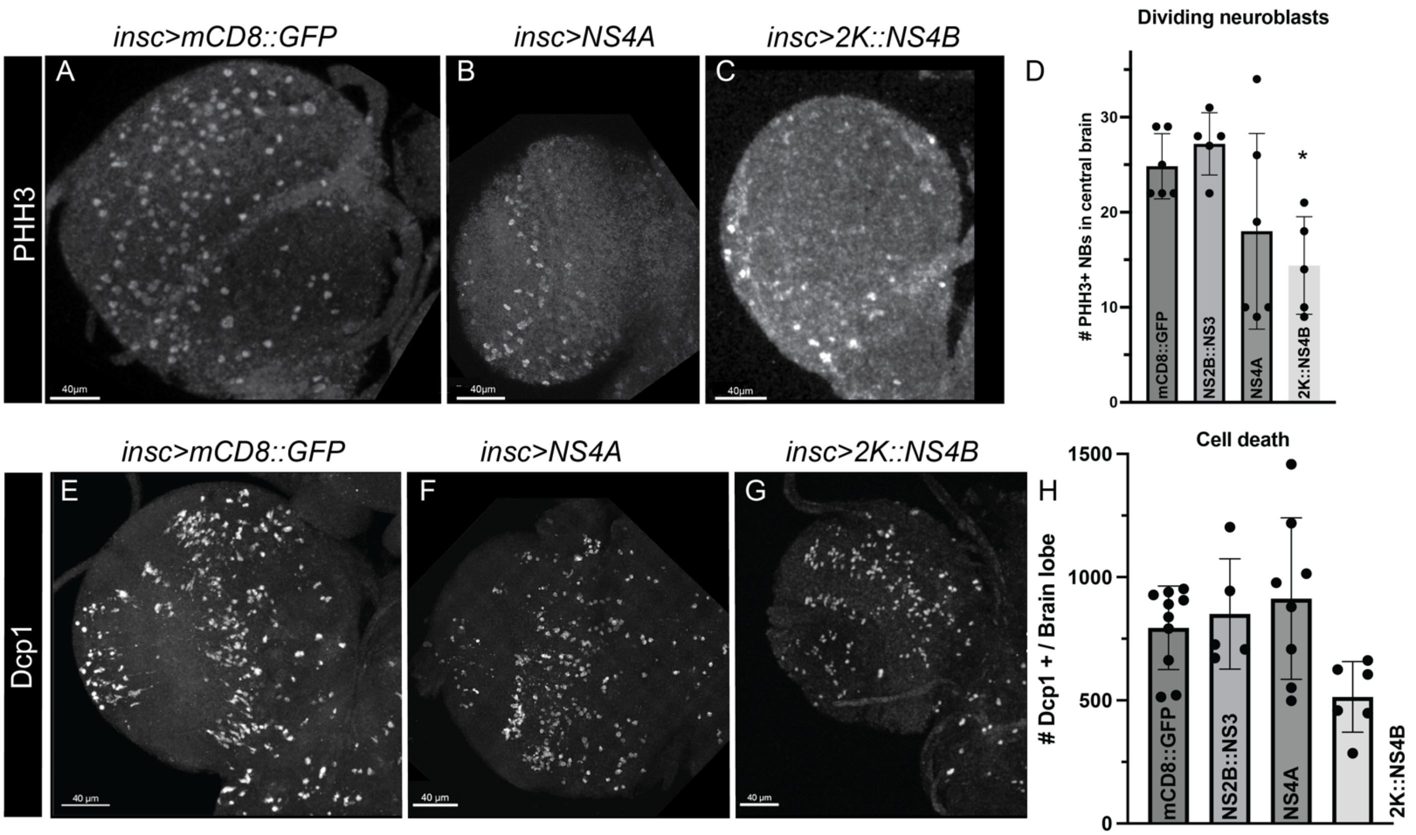

Fig. S2. 2K::NS4B reduces neuroblast division in the developing brain. (A-D) 3rd instar brains from animals expressing (A) mCD8::GFP (control), (B) NS4A, or (C) 2K::NS4B stained for phospho-histone H3 (PHH3, white) which marks dividing cells. The number of PHH3 positive neuroblasts in each central brain region is plotted in (D). 2K::NS4B significantly reduced PHH3 central brain neuroblasts (One way ANOVA, $p=0.05$ compared to mCD8-GFP). (E-H) 3rd instar brains from animals expressing (A) mCD8::GFP (control), (B) NS4A, or (C) 2K::NS4B, stained for activated Dcp1 (white) which indicates active *Drosophila* caspase 1 and is an indicator of apoptosis. No significant differences were found, but a trend of increased cell death with NS2B:NS3 and NS4A was noted. One way ANOVA with multi-comparisons posttest. For all images, an 8 μm stack at the same region is shown.

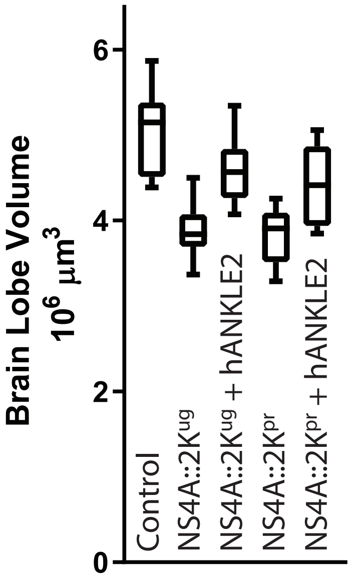

Fig. S3. Human ANKLE2 rescues NS4A::2K induced defects from both Puerto Rican and Ugandan strains. Brain volume of third instar larvae with expression of NS4A::2K^{ug} (Uganda) or NS4A::2K^{pr} (Puerto Rico) with or without wild type human *ANKLE2*, which rescues NS4A::2K induced microcephaly.

Disease Models & Mechanisms • Supplementary information

Table S1. A list of all ZIKV transgenic lines produced. List includes backbone vector and attP site.

ZIKV transgenic lines produced
pGW-C-HA.attB_VK37
pGW-NS1-HA.attB_VK37
pGW-NS2A-HA.attB_VK37
pGW-NS4A-HA.attB_VK 37
pGW-NS4B-HA.attB_VK 37
pGW-NS5-HA.attB_VK 37
pGW-prM::E-HA.attB_VK37
pGW-NS2B::NS3-HA.attB_VK37
pGW-NS4A::2k-HA.attB_VK37
pGW-2K::NS4B-HA.attB_VK37
pGW-NS1[cam V188A]-HA.attB_VK37
pGW-prM::E[cam N139S]-HA.attB_VK37

Table S2. Lethal stage of ZIKV crosses. GAL4 driver line and transgenic lines are noted. If animals were lethal prior to eclosion, the lethal stage is noted. ND = not determined.

GAL4	C	prM::E	NS1	NS2A	NS2B::NS3	NS4A	NS4A::2K	2K::NS4B	NS4B	NS5
Act	-	pupal	L1	-	-	L2	-	embryonic	embryonic	ND
Tub	-	ND	ND	-	-	before L3	pupal	early L3	before L3	ND
da	-	-	escapers	-	-	L1	pupal	embryonic	L3	L2
pnr	-	-	ND	-	-	escapers	-	embryonic	embryonic	ND
GMR	-	-	-	-	-	-	-	-	-	-

Disease Models & Mechanisms • Supplementary information

Table S3. Number of animals in an example ZIKV cross. One experiment using *ey-GAL4* to drive expression of ZIKV proteins and the number of animals assessed each day is noted. Each row represents a new day. The total number of animals assessed is in the last row**.** Each experiment was conducted in a similar manner.

GMR-GAL4	prE::M	NS2A	NS1	NS2B::NS3	NS4A	NS4A::2K	2K::NS4B	NS4B	NS5
	12	41	12	25	27		22		
	18	12	4	11	5		2		
	11	8	10	11	27		8		
	6	12	49	7	4		16		
	16	7	52	6	33		7		
	18	4	36	2	45		15		
	55	20	13	2	54		14		
	49	15	60	7	49		69		
	4	28	3	20	8		12		
	41	15	63	4	40		6		
	13	18	30	17	8		62		
	10	2	105		13		13		
	4		13		9		48		
	4		10		9				
Total N	261	182	460	112	331	0	294	0	0

Disease Models & Mechanisms • Supplementary information
